# Target RNA modification for epigenetic drug repositioning in neuroblastoma: computational omics proximity between repurposing drug and disease

**DOI:** 10.18632/aging.103671

**Published:** 2020-10-12

**Authors:** Pei Ma, Lifeng Yue, Sen Zhang, Dacheng Hao, Zhihong Wu, Lijia Xu, Guanhua Du, Peigen Xiao

**Affiliations:** 1 Institute of Medicinal Plant Development, Chinese Academy of Medical Sciences, Peking Union Medical College, Beijing 100193, China; 2 State Key Laboratory of Bioactive Substance and Function of Natural Medicines, Institute of Materia Medica, Chinese Academy of Medical Sciences and Peking Union Medical College, Beijing 100050, China; 3 Department of Neurology, Dongzhimen Hospital, Beijing University of Chinese Medicine, Beijing 100700, China; 4 Biotechnology Institute, School of Environment and Chemical Engineering, Dalian Jiaotong University, Dalian 116021, China; 5 Department of Orthopedics, Peking Union Medical College Hospital, Chinese Academy of Medical Sciences and Peking Union Medical College, Beijing 100730, China

**Keywords:** neuroblastoma, drug repurposing, gene expression, the connectivity map, L1000 FWD

## Abstract

RNA modifications modulate most steps of gene expression. However, little is known about its role in neuroblastoma (NBL) and the inhibitors targeting it. We analyzed the RNA-seq (n=122) and CNV data (n=78) from NBL patients in Therapeutically Applicable Research to Generate Effective Treatments (TARGET) database. The NBL sub-clusters (cluster1/2) were identified via consensus clustering for expression of RNA modification regulators (RNA-MRs). Cox regression, principle component analysis and chi-square analysis were used to compare differences of survival, transcriptome, and clinicopathology between clusters. Cluster1 showed significantly poor prognosis, of which RNA-MRs’ expression and CNV alteration were closely related to pathologic stage. RNA-MRs and functional related prognostic genes were obtained using spearman correlation analysis, and queried in CMap and L1000 FWD database to obtain 88 inhibitors. The effects of 5 inhibitors on RNA-MRs were confirmed in SH-SY5Y cells. The RNA-MRs exhibited two complementary regulation functions: one conducted by TET2 and related to translation and glycolysis; another conducted by ALYREF, NSUN2 and ADARB1 and related to cell cycle and DNA repair. The perturbed proteomic profile of HDAC inhibitors was different from that of others, thus drug combination overcame drug resistance and was potential for NBL therapy with RNA-MRs as therapeutic targets.

## INTRODUCTION

Neuroblastoma (NBL) is a clinical heterogeneous malignancy of the peripheral nervous system, which accounts for ~15% of all childhood cancer death [1]. The deregulation development of neural crest, a stem/progenitor cell population, is considered critical in NBL initiation [2, 3]. Nowadays patients with severe NBL still have poor survival rates (<50%) despite intensive multimodality therapy [4]. Therefore, there is an urgent need to identify NBL vulnerabilities for potential pharmacological intervention with more efficiency and less toxicity.

Epigenetic modification of RNA modulates most steps of gene expression, including DNA transcription, post-transcriptional RNA modifications, and mRNA translation [5, 6]. Four RNA modifications regarding methylation or isomerization, have been reported abundant and important for differentiation of neural progenitor cells and neurological diseases: 5-methylcytosines (m5C), 5-hydroxymethylcytosine (hm5C), adenosine to inosine editing (A-I editing), and N6-methyladenosine (m6A) [7], whereas limited study reports their functions in NBL. RNA modification regulators (RNA-MRs) are three kinds of proteins involved in modification: methyltransferase (writer, W), demethylase (eraser, E), and RNA-binding protein (reader, R) [8]. The m5C is methylated by NSUN2 or TRDMT1 (formerly DNMT2), read by ALYREF, and oxidized to hm5C by TETs [9]. The A-I editing is catalyzed by ADAR, ADARB1, and ADATs [10]. The m6A is catalyzed by a multiprotein complex containing enzymes (METTL3 and METTL14) and accessory proteins (VIRMA, ZC3H13, WTAP, and RBM15), and demethylases by FTO and ALKBH5 [11]. The RNA-MRs regulate expressions of tumor driver/suppressor genes, and are functionally linked to survival, proliferation, differentiation, invasion and drug resistance of tumor cells. Recently the crosstalk between RNA modifications is also reported to provide a potential exquisite functional control [8, 12]. Therefore RNA-MRs emerge as important regulators, prognostic markers and therapeutic targets in cancer [8].

Currently no therapeutic target on RNA modification is clinically available, despite our ever-growing understanding of its biology. Development strategies for epigenetic drug targeting DNA modification support a reference as following: 1) inhibitors for a targetable enzymatic activity and associated binding pockets, 2) antagonists for reader proteins, and 3) inhibitors of downstream or upstream effectors for undruggable or tumor-suppressing regulators [9, 10]. However, our understanding of structures of RNA-MRs remains incomplete, and developing a brand-new drug consumes an enormous amount of time, money, and effort. Therefore, it is a cost-effective way to develop a computational drug repositioning approach, which predicts potential known drugs targeting RNA-MRs in NBL [13]. These drugs encompass potential enzyme inhibitor, reader antagonists, and inhibitors of down-stream or upstream effectors.

Many commonly used traditional drug databases, such as DrugBank, ChEMBL, and ZINC, are developed to predict potential drug–target associations on a large scale. Data for these databases come from the ‘one molecule-one target-one disease’ paradigm, which identify effective compounds that affect specific proteins [14]. However, the major limiting factor is that pharmaceuticals are designed to target individual factors in a disease system, but complex diseases are multifactorial in nature and vulnerable at multiple attacks. Thus, in this study, we use a state-of-the-art computational omics proximity strategy [15], which is recently developed for drug repositioning. We integrate the “omics” data at transcriptional and proteomic levels, which are obtained by L1000 platform and liquid chromatography-mass spectrometry, respectively. Due to a high degree of statistical dependencies between gene expression, the L1000 technology uses a computational model for 978 landmark genes to capture most of the information contained within the entire transcriptome [16]. Many databases and web servers are developed based on data obtained by L1000 technology, such as Connectivity Map (CMap) and L1000 fireworks display (L1000FWD) databases. L1000FWD provides interactive visualization of L1000 profiles of 12716 genes from 20449 compounds in 68 cell lines [17]. It supplied transcriptome changes for single compound only. Meanwhile, CMap provides L1000 profiles of 12328 genes from 42,080 perturbagens (19,811 small molecule compounds, 18,493 shRNAs, 3,462 cDNAs, and 314 biologics) in 77 cell lines [16]. Besides transcriptome changes, CMap integrates perturbagen-induced proteomics data regarding phosphorylation changes (P100) and histone modifications (GCP) [15]. Therefore, CMap facilitates the overall functional mapping, whereas limits query input for optimization. Here we query both CMap and L1000FWD to obtain an optimized global profile of the drug perturbation. However, there is a main technical limitation for this study. Data in CMap and L1000FWD are generated in cell lines, which leads a possible different from that in clinic.

Here, by focusing directly on human data, we firstly used a series of computational models to confirm a core prognostic gene set containing RNA-MRs and function-related genes. This included: 1) clustering in NBL patients according to RNA-MRs’ expression; 2) difference evaluation between sub-clusters for transcriptome profile, copy number variation (CNV) profile, gene function, survival and clinicopathology; 3) prognostic values of RNA-MRs and their downstream or upstream related genes. Then we calculated omics proximity between core prognostic gene set and data in L100FWD and CMap. This included: 1) reasonability evaluation of core prognostic gene set as a reduced representation of differentially expressed genes (DEGs); 2) drug candidates in CMap and L1000 FWD via similarity query for core prognostic gene set; 3) proteomic profiles and chemical structure characteristics of drug candidates; 4) drug response biomarker network for confirmation signal or combination repurposing of drug candidates. Finally, we performed biological experiments to confirm 7 predicted drug candidates. Further, omics proximity strategy could also be used for predicting drug candidates for treating other diseases.

## RESULTS

### Current RNA-seq sample and CNV sample was suitable for information retrieval in NBL patients

**Figure 1:**
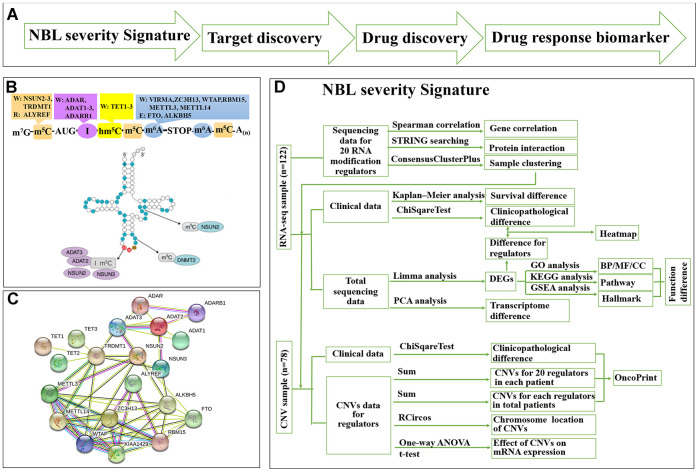
**Summary for schematic diagram and RNA modification regulators (RNA-MRs) in NBL.** (**A**) The schematic diagram of target mining and drug screening in this study. (**B**) A schematic view of location and types of predominant RNA modification. W: writer (methyltransferase), R: reader (RNA-binding protein), E: eraser (demethylase). TRDMT1: formerly DNMT2. (**C**) The protein interaction network among RNA-MRs using STRING analysis. KIAA1429: VIRMA. (**D**) The detailed schematic diagram for determination of NBL severity signature.

We used a ‘NBL severity signature—target discovery—drug discovery—drug response biomarker’ process to predict repositioning drug (Figure 1A). We collected a list of 20 RNA-MRs regarding differentiation of neural progenitor cells and neurological diseases (Figure 1B), which had functional relationship (Figure 1C). The contributions of 20 RNA-MRs on NBL severity signature was studied as Figure 1D.

The age, International Neuroblastoma Staging System (INSS) stage, MYCN gene amplification, DNA ploidy, histology, MKI (mitosis karyorrhexis index), and Children Oncology Group (COG) risk score were commonly used as prognostic indexes in clinic for evaluating NBL severity. Three datasets were retrieved from Therapeutically Applicable Research to Generate Effective Treatments (TARGET) program and The Cancer Genome Atlas (TCGA) network: (1) the clinical dataset containing clinicopathological information of 834 NBL patients; (2) the RNA-seq dataset containing transcriptome data and clinicopathological information of 122 NBL patients, and (3) the CNV dataset containing copy number variation data (obtained by whole genome sequencing) and clinicopathological information of 78 NBL patients (Supplementary Figure 1D). To test the reproducibility of patients with RNA-seq/CNV data for overall NBL patients, clinicopathological information and their association with overall survival for 3 datasets is compared. Compared with total patients, RNA-seq dataset contained older patients with higher mortality, COG risks, unfavorable histology, and advanced INSS stage, while CNV dataset contained patients with more significantly amplification of *MYCN* gene, (Table 1, P<0.05). These indexes showed similar prognostic patterns among three groups (Supplementary Figure 1A–1C). Information retrieval from RNA-seq and CNV data was thus suitable for NBL patients.

**Table 1:** Clinicopathological features of patients in this study.

		**Clinical Sample**		**RNA-seq Sample**		**CNV Sample**	
		**Number**	**Percentage**	**Number**	**Percentage**	**P value**	**Number**	**Percentage**	**P value**
Total		834	100.00%	122	100.00%		78	100.00%	
Age (years)	>10	18	2.16%	4	3.28%	0.004	2	2.56%	0.285
	>5	71	8.51%	21	17.21%		15	19.23%	
	>3	199	23.86%	33	27.05%		19	24.36%	
	>2	170	20.38%	22	18.03%		11	14.10%	
	>1	164	19.66%	19	15.57%		9	11.54%	
	≤1	212	25.42%	23	18.85%		22	28.21%	
Gender	female	357	42.81%	47	38.52%	0.372	28	35.90%	0.281
	male	477	57.19%	75	61.48%		50	64.10%	
Fustat	alive	557	66.79%	65	53.28%	0.003	50	64.10%	0.294
	dead	277	33.21%	57	46.72%		28	35.90%	
Futime (years)	<=3	246	29.50%	44	36.07%	0.614	20	25.64%	0.545
	<=5	215	25.78%	21	17.21%		15	19.23%	
	<=10	311	37.29%	48	39.34%		40	51.28%	
	>10	62	7.43%	9	7.38%		3	3.85%	
INSS	stage1	80	9.59%	0	0.00%	<0.001	0	0.00%	0.132
	stage2	58	6.95%	1	0.82%		1	1.28%	
	stage3	86	10.31%	6	4.92%		6	7.69%	
	stage4	561	67.27%	97	79.51%		54	69.23%	
	stage4s	49	5.88%	18	14.75%		17	21.79%	
COG	Low	152	18.23%	12	9.84%	0.006	11	14.10%	0.541
	Intermediate	105	12.59%	11	9.02%		11	14.10%	
	High	577	69.18%	99	81.15%		56	71.79%	
MYCN	Not Amplified	597	71.58%	94	77.05%	0.208	65	83.33%	0.033
	Amplified	237	28.42%	28	22.95%		13	16.67%	
Ploidy	Hyperdiploid	536	64.27%	69	56.56%	0.099	49	62.82%	0.806
	Diploid	298	35.73%	53	43.44%		29	37.18%	
Histology	Favorable	253	30.34%	25	20.49%	0.025	22	28.21%	0.703
	Unfavorable	581	69.66%	97	79.51%		56	71.79%	
Grade	Differentiating	54	6.47%	10	8.20%	0.478	4	5.13%	0.81
	Undifferentiated	780	93.53%	112	91.80%		74	94.87%	
MKI	Low	338	40.53%	48	39.34%	0.844	30	38.46%	0.906
	Intermediate	251	30.10%	42	34.43%		26	33.33%	
	High	245	29.38%	32	26.23%		22	28.21%	

Mann-Whitney U test was used for comparison between RNA-seq and total clinical samples, as well as between CNV and total clinical samples.

Undifferentiated: Undifferentiated or Poorly Differentiated.

### Differenced expression of RNA-MRs defined two NBL clusters with different survival and clinicopathology

**Figure 2:**
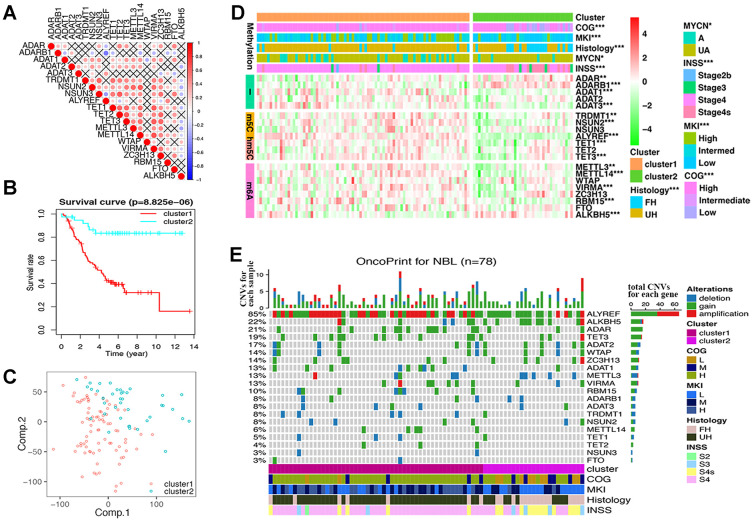
**Differences of gene expression and CNV profile, survival and clinicopathological features in two groups defined by RNA modification regulators (RNA-MRs).** (**A**) Spearman correlation analysis of RNA-MRs. Red: positive relation of gene expression between two genes; blue: negative relation. Color scale: the degree of relation. Cross (×): *P*>0.05 (no significant difference). (**B**) Kaplan–Meier overall survival curves for NBL patients in two groups. Ordinal: the percentage of survival; abscissa: survival years. "+" marks in line: censoring samples. (**C**) Principal component analysis of the transcriptome expression profile in the space of the first two principal components (Comp.). (**D**) Heatmap comparison of RNA-MRs for two groups. Horizontal color stripe above heatmap: one clinicopathological feature per line. Vertical color stripes: different methylation type regulated by each RNA-MR. Heatmap: expression differences of RNA-MRs (gradient color from green to red in each line showed downregulated to upregulated levels of each gene). *P < 0.05, **P < 0.01, and ***P < 0.001 between Cluster1 and Cluster2. (**E**) The OncoPrint of CNV pattern in two groups. Blue dot: deletion; green dot: gain; red dot: amplification. The upper barplot: the number of CNVs per patient; the right barplot: the number of genetic mutations per gene (corresponding rate was at left); the bottom color stripe: clinicopathological features for each patient.

Twenty RNA-MRs had strong correlation at RNA expression level in NBL patients (Figure 2A). Thus, they shared similar functions in NBL disease course according to the ‘Guilt-by-Association’ principle [18]. The k-means clustering was a most commonly used centroid model in cancer study to detect subtypes, which was useful for prognosis prediction and personalized treatments [19]. As shown in Supplementary Figure 2A, RNA-seq samples could be clustered into 2~5 sub-clusters, with clustering stability increasing from k = 2 to 10. The performance of 2/3/4/5-cluster were quantified using adjusted Rand index (ARI), which evaluated the similarity between the resulting partition and the gold standard partition [20]. Here, two survival state (alive or dead) were assumed to be gold standard partition, and ARI were 0.136/0.076/0.086/0.059 for 2/3/4/5-cluster. Interestingly, the overall clustering performance for 2-cluster was high while the clustering stability was relatively low, compared with 3/4/5-cluster. This showed that a simple classification problem is not necessarily an easy clustering problem. Meanwhile, cluster1 in 2-cluster seemed to have a more significantly poor prognosis (*P*= 8.825×10^-6^ *vs.* 9.028×10^-4^/1.485×10^-4^/6.387×10^-4^ in 3/4/5-cluster), and a clear distinct transcriptional landscape in 2-cluster could be seen using principal components analysis (PCA) (Figure 2B, 2C and Supplementary Figure 2B). Thus, we chose 2-cluster as representative classification for NBL severity. As shown in Figure 2D, cluster1 showed upregulated A-I editing in tRNA modification, m5C, hm5C, and m6A, together with downregulated A-I editing in mRNA modification (P<0.01). And cluster1 was also significantly correlated with higher COG risks, higher MKI, unfavorable histology, *MYCN* gene amplification and advanced INSS stage (P < 0.05, Table 2).

**Table 2:** Clinicolpathological features are different between two groups in RNA-seq and CNV dataset.

		**RNA-seq Sample**			**CNV Sample**		
		**cluster1**	**cluster2**	**P value**	**cluster1**	**cluster2**	**P value**
Total		83	39		53	25	
INSS	stage1	0	0	0.0001	0	0	9.41E-05
	stage2	1	0		1	0	
	stage3	2	4		2	4	
	stage4	75	22		45	9	
	stage4s	5	13		5	12	
COG	Low	2	10	3.47E-06	2	9	4.01E-05
	Intermediate	5	6		5	6	
	High	76	23		46	10	
MYCN	Not Amplified	59	35	0.0399	42	23	0.2779
	Amplified	24	4		11	2	
Histology	Favorable	8	17	4.27E-05	7	15	5.92E-05
	Unfavorable	75	22		46	10	
MKI	Low	23	25	0.0004	14	16	0.0026
	Intermediate	32	10		19	7	
	High	28	4		20	2	

These results showed that dysregulated expression of RNA-MRs strongly reflected the changes of total transcriptome, and the disease severity as well. Dysregulated RNA-MRs predicted poor patient prognosis, independently of other prognostic markers such as *MYCN* amplification status, age at the time of diagnosis, and disease stage.

### CNVs of RNA-MRs were associated with their expression

CNV samples belonging to cluster1 was also significantly correlated with higher COG risks, higher MKI, unfavorable histology, and advanced INSS stage (P < 0.0001, Figure 2E and Table 2).

**Figure 3:**
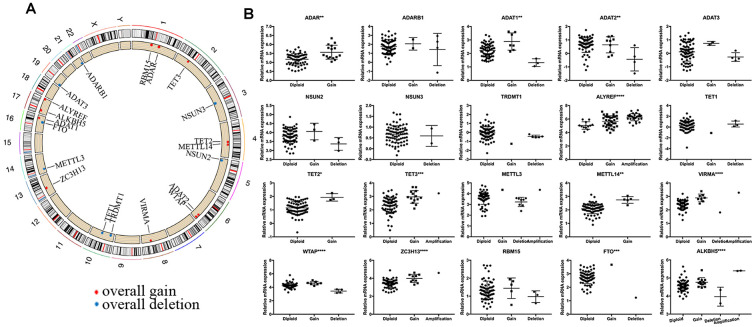
**Summary of CNV region and correlation between CNV pattern and gene expression.** (**A**) Circos plot illustrating CNV regions for RNA modification regulators (RNA-MRs). Outermost circle: chromosomes; inner circle: the main type and location of CNVs (red: gain, blue: deletion). (**B**) Correlation between CNV patterns and expression levels. Ordinal: the relative gene expression levels; abscissa: CNV types. * P < 0.05, ** P < 0.01, and *** P < 0.001 among different CNV types.

We observed high CNVs of RNA-MRs in genome level of NBL. In detail, the m5C “reader” ALYREF had the highest frequency of CNV events (85%), followed by the m6A “eraser” ALKBH5 (22%), both of which were located in chromosome 17 (Figure 2E and 3A). The amplification of ALYREF was the most frequent alteration in cluster1, and the simultaneous gain of ALYREF and ALKBH5 ranked first in cluster2, implying the importance of CNVs on dysregulated RNA modification network and NBL severity.

The effects of CNVs of RNA-MRs on their mRNA expression were further confirmed. Results showed that copy number gain or amplification were related to higher mRNA expression, while deletion resulted in a decline of mRNA expression (Figure 3B). CNVs in drug targets may change their abundance and drug-binding pocket of receptors [21], thus the high frequency of CNVs for RNA-MRs here made inhibitors for their enzymatic activity and antagonists for reader proteins more easily to be resistance.

### Differences of functional annotation in two NBL groups

**Figure 4:**
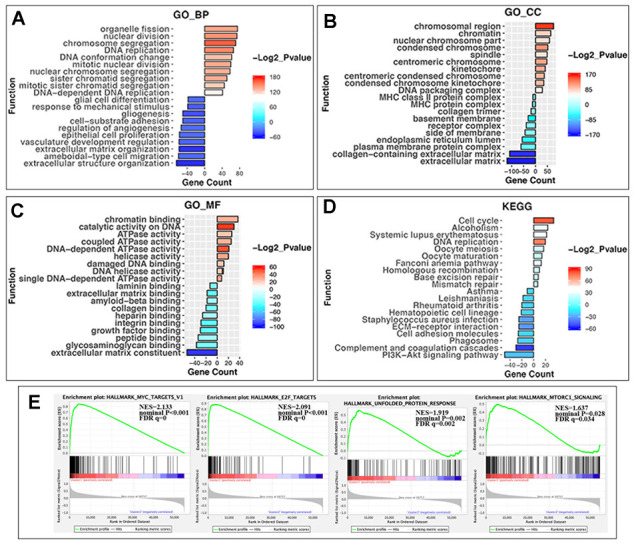
**GO, KEGG pathway enrichment analysis and the gene set enrichment analysis (GSEA) of differentially expressed genes (DEGs) in two groups.** (**A**) Biological process of GO analysis; (**B**) Cellular component of GO analysis; (**C**) Molecular function of GO analysis; (**D**) KEGG pathways analysis. Y-axis: name of enrichment item; X-axis: gene count in each item (positive value for cluster1 and negative for cluster2); gradient color of the bar chart: -Log2_Pvalue (values >0 for -Log2_Pvalue in Cluster1, and values <0 for -(-Log2_Pvalue) in Cluster2). (**E**) GSEA enrichment plots in HALLMARK datasets from left to right: MYC targets, E2F targets, unfolded protein response, and MTORC1 signaling. In each plot, top panels: enrichment score (ES) for each gene; bottom panels: the ranking metrics of each gene; Y-axis: ranking metric values; X-axis: ranks for all genes. NES: normalized ES. Norm P (nominal p value): the statistical significance of the observed ES for this plot. FDR q value: a significantly enriched enrichment plot if q< 0.25.

The above findings suggested that the clustering NBL according to gene expression of RNA-MRs was closely correlated to the malignancy of NBL. Then DEGs and functional differences between groups were evaluated. Altogether, 959 and 1154 genes were respectively upregulated and downregulated significantly (log2 (fold change) >1 or <-1, P < 0.05 and the false discovery rate (FDR) <0.05) in cluster1, which showed more severe disease course. In cluster1, DEGs enriching in biological process (BP) of GO (gene ontology) were predominantly associated with malignancy-related biological processes, including cell proliferation, extracellular matrix organization, angiogenesis, and migration (Figure 4A). The DEGs in GO cellular component (CC) were enriched in chromosome part, cytoplasmic membrane and extracellular matrix (Figure 4B). And DEGs in GO molecular function (MF) were associated with chromatin binding, catalytic activity on DNA, and ATPase activity (Figure 4C). Similar changes in corresponding signaling pathways were also observed in Kyoto Encyclopedia of Genes and Genomes (KEGG) pathway analysis (Figure 4D).

Furthermore, gene set enrichment analysis (GSEA) revealed that the malignant hallmarks of tumors, including MYC targets (NES=2.13, nominal P < 0.001), E2F targets (NES=2.09, nominal P < 0.001), unfolded protein response (NES=1.92, nominal P = 0.002) and MTORC1 signaling (NES=1.64, nominal P=0.028), were significantly associated with cluster1 (Figure 4E).

All of these findings indicated that the dysregulated expression of RNA-MRs was closely correlated with the malignancy of NBL.

### Mapping the NBL signature onto drug-perturbed transcriptome profiles in L1000FWD

**Figure 5:**
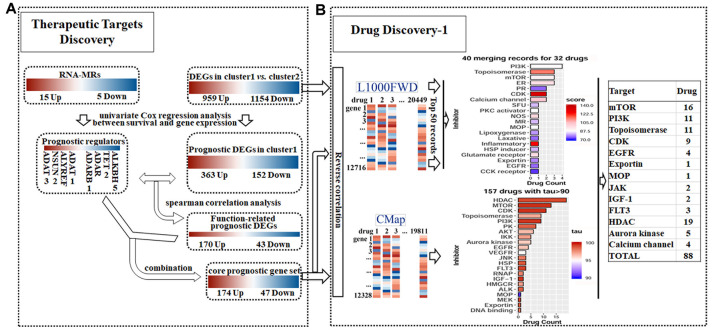
Detailed chart for therapeutic targets discovery (**A**) and drug discovery (**B**). (**A**) The prognostic potential of differentially expressed genes (DEGs) and RNA modification regulators (RNA-MRs) in cluster1 (severe NBL) were analyzed. Genes correlated to survival formed prognostic DEGs set and prognostic RNA-MRs set, respectively. Then function-related prognostic DEGs were identified from prognostic DEGs as genes positively related to prognostic RNA-MRs. The function-related prognostic DEGs and prognostic RNA-MRs together formed core prognostic gene set. (**B**) The reversed similarities of gene expression pattern in DEGs and core prognostic gene set were calculated in L1000FWD respectively. The same records in two searches were integrated according to target type. The reversed similarities of core prognostic gene set only was calculated in CMap. Then inhibitors from L1000FWD and CMap were summarized according to target type. PR: progesterone receptor; SFU: sodium fluorescein uptake; MR: Mineralocorticoid receptor; MOP: Mitochondrial oxidative phosphorylation; RNAP: RNA polymerase.

To evaluate core prognostic gene set as a reduced representation of DEGs, the top 50 records with opposed expression profiles of DEGs and core prognostic gene set were collected (Supplementary Figure 3). These records had similarity scores ranging from 0.090 to 0.141, and from 0.303 to 0.483 (P<0.0001), respectively (Supplementary Tables 1 and 2). Among them 40 records for 32 drugs were overlapped for two queries, implying the representativeness of core prognostic gene set for DEGs and disease severity (Figure 5B). And 11 overlapped drugs were previously reported in NBL cells or clinic. Targets for top 50records were mainly PI3K, topoisomerase, mTOR, estrogen receptor, and CDK. Besides that, reversed expression of RNA-MRs was queried in L1000FWD. Topoisomerase inhibitors, such as amsacrine and idarubicin, were top records with relatively high similarity scores (Supplementary Table 3).

### Mapping the NBL signature onto drug-perturbed transcriptome profiles in CMap

The opposed expression profile of core prognostic gene set was queried in CMap. There were 157 compounds with tau> 90 (Supplementary Table 4). Together with Perturbagen Class (PCL) analysis, these compounds were enriched in HDAC inhibitor, PI3K inhibitor, mTOR inhibitor, topoisomerase inhibitor, and CDK inhibitor (Figure 5B and Supplementary Table 5).

### Overlapped drugs in CMap and L1000FWD

**Figure 6:**
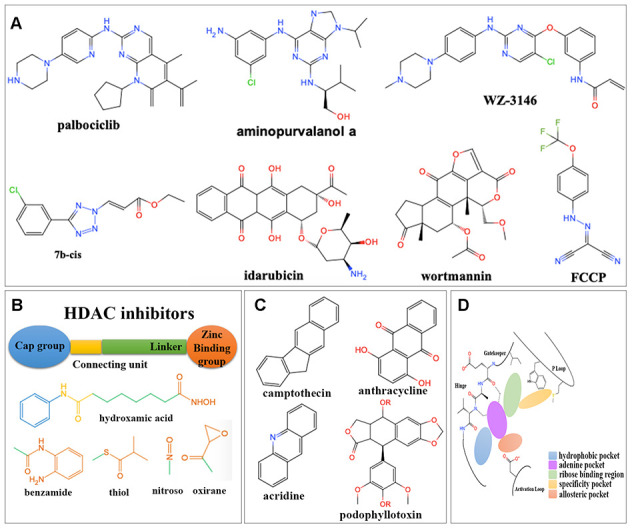
**Structures for different kinds of inhibitors.** (**A**) The structure of 7 overlapped drugs in CMap and L1000FWD. (**B**) Core structures for HDAC inhibitors. (**C**) Core structures for topoisomerase inhibitors. (**D**) Core structures for kinase inhibitors.

There were totally 88 potential drugs selected from CMap or L1000FWD for further analysis (Figure 5B). Seven of them were obtained in both CMap and L1000FWD query, namely, palbociclib, wortmannin, FCCP, idarubicin, aminopurvalanol-a, 7b-cis, and WZ-3146 (Figure 6A). They belonged to the following target classes: CDK, PI3K, topoisomerase, EGFR, mTOR, exportin, and mitochondrial oxidative phosphorylation uncoupler (Supplementary Table 6). Palbociclib, wortmannin, FCCP, WZ-3146, and idarubicin were reported to inhibit cell cycle and vascularization in clinic or neuroblastoma cells, which meant our results are convinced [22, 23]. Purvalanol A was used in clinic for NBL [24]. Aminopurvalanol A, a homolog of purvalanol a, reversed core prognostic gene expression, seemed to be a choice for NBL via targeting CDK [25]. Exportin 1 (XPO1) was a nuclear receptor exporter involved in the active transport of transcription factors, tumor suppressor proteins, cell-cycle regulators and RNA molecules [26]. The 7b-cis, inhibitor of XPO1, showed significant therapy potential in both CMap and L1000FWD, was worth for further study [27].

### The MOA and structure analysis for potential inhibitor classes

Among 88 potential predicted drugs, HDAC inhibitors were the largest class. There was a well-admitted pharmacophore model illustrating the structure of HDAC inhibitors (Figure 6B), which consisted of a zinc binding group (ZBG), a linker chain mimicking the lysine side chain, a connecting unit, and a terminal functional “cap” group interacting with the external surface [28]. The ZBGs of most HDAC inhibitors predicted here were hydroxamic acids.

DNA topoisomerase inhibitors were one of the largest predicted classes, which adjusted DNA’s topological structure by cutting, transferring and re-connecting with DNA chains [29]. Here inhibitors for both Topoisomerase I and Topoisomerase II were obtained, which were mainly camptothecin derivatives and anthracyclines (Figure 6C), respectively.

Protein kinases (PKs) were important for signal transduction from extracellular to intracellular, signaling cascade and finally cell response, such as cell proliferation, differentiation, and inflammatory [30]. The preserved hinge region of ATP binding site [31] in all kinases led to the structural similarity of kinase inhibitors (Figure 6D). Inhibitors usually contained a hinge-binding motif, such as a morpholinyl or purinyl substituent, to occupy the adenine binding site of ATP. Hydrophobic pockets were commonly occupied by tolyl group. Allosteric pockets were commonly occupied by terminal piperazinyl-phenyl group, or long-chain such as trifluoromethyl group, which had a hydrophilic group exposing to the solvent region [32]. For kinase consists of “specificity” pocket, such as P-loop in JAK, the terminal group extended into a cleft underneath the P-loop in the N-lobe [33].

Two kinds of PK inhibitors, serine-threonine kinase inhibitors and tyrosine kinase inhibitors, were mainly predicted here. There were four kinds of serine-threonine kinase inhibitors. The mTOR inhibitors were mainly macrolides, morpholino-pyrimidine-phenyl derivatives, and quinoline-imidazoquinoline-phenyl derivatives. The PI3K inhibitors were commonly dual inhibitors and shared similar structures with mTOR inhibitors. The CDKs inhibitors were mainly phenylamino-purine derivatives. Aurora kinase inhibitors were commonly phenylamino-pyrimidine-phenyl derivatives. There were one kind of non-receptor tyrosine kinase (JAK) inhibitor and three kinds of receptor tyrosine kinase inhibitors (FLT3, EGFR and IGF-1) predicted here. JAK inhibitors were triterpenoid and indolone derivatives. FLT3 inhibitors were pyrimidine and staurosporine derivatives. EGFR inhibitors were pyrimidine, quinoline, and quinazoline derivatives. IGF-1 inhibitors were benzimidazole and pyrrolotriazine derivatives (Supplementary Table 6).

### Proteomic profile of drugs targeting different targets in CMap

**Figure 7:**
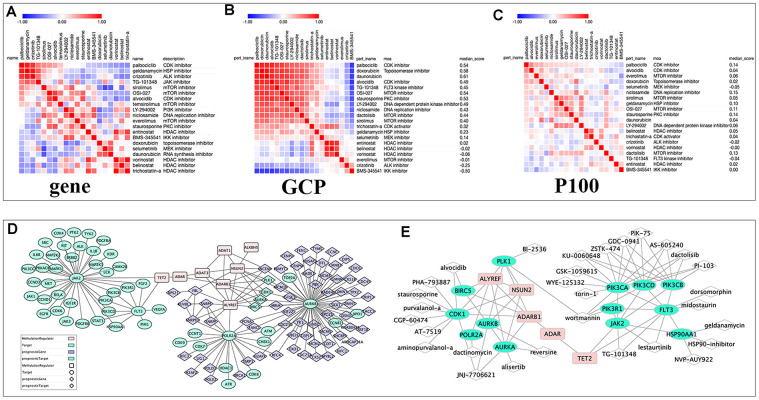
**The relationships among drugs, core prognostic genes, and targets.** (**A**–**C**) The similarity matrix of genomic, proteomic connectivity for compounds with tau >95 in CMap (**A**), GCP (**B**) and P100 (**C**) database. The map was sorted with the strongest perturbagen connections at the top in red. (**D**) Schematic representation of the association between RNA modification regulators (RNA-MRs), function-related prognostic DEGs and target of compounds with tau>95. Pink squares: RNA-MRs; green cycles: targets; blue diamond: prognostic DEGs; green diamond: prognostic genes as targets. (**E**) Schematic representation of targets with a direction relationship with RNA-MRs, together with corresponding inhibitors.

Fifteen compounds with tau> 95 could be find in P100 and GCP Proteomics Connectivity Hubs, and their genomic and proteomic connectivity were shown as similarity matrix (Figure 7A–7C). There were strong expression connections between CDK inhibitors, MTOR inhibitors and topoisomerase inhibitors, and negative connections between them and HDAC inhibitors in genomic and proteomic perturbed profiles.

### The relationship between targets, function-related prognostic genes and RNA modification regulators

The drug-perturbed profiles at gene and protein levels were clustered into two groups by their connections, which implied there were not only one potential role for RNA-MRs. To confirm this hypothesis, we construct network between drugs, targets, prognostic genes and RNA-MRs (Figure 7D, 7E). Figure 7D showed these genes were clustered into two groups. One cluster was mainly regulated by TET2 and enriched in JAK and PI3K signaling pathways. The other cluster was mainly regulated by NSUN2/ALYREF/ADAR regulators, and enriched in cell cycle and DNA replication process. In Figure 7E, sub-cluster at left was genes enriched in CDK, topoisomerase, MTOR related signaling. They had connections with ALYREF, NSUN2, and ADARB1 via PLK1, BIRC5, CDK1, AURKA. These genes were both drug targets and prognostic genes, which had closely relationship with RNA-MRs. The other sub-cluster at right showed genes enriched in HDAC, JAK, MEK related signaling. They had connections with TET2 via JAK2 and FLT3. Thus, there were two enriched functions of core prognostic genes, and combined use of different inhibitors may be better to reverse expression profile and block disease course.

### Experimental validation of the predicted drugs in neuroblastoma SH-SY5Y cells

**Figure 8:**
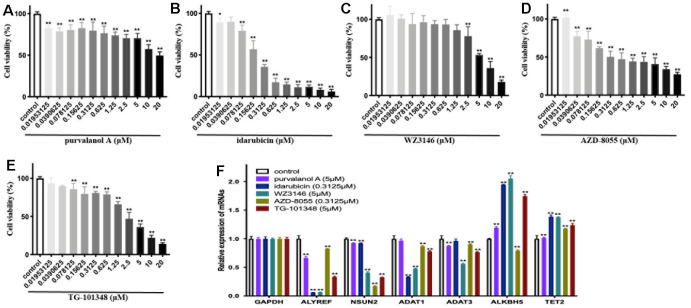
**The cellular viability and dysregulation of RNA modification regulators (RNA-MRs) in human neuroblastoma cell line SH-SY5Y cells.** (**A**–**E**) Concentration-dependent inhibition of cellular viability by purvalanol A, idarubicin, WZ3146, AZD-8055, TG-101348 (10^-8^~10^-5^ mol/L) at 24 h, measured by CCK8 assay. (**F**) The expression levels of ALYREF, ADAT1, ADAT3, NSUN2, ALKBH5, TET2 detected by quantitative real-time PCR. *p < 0.05, **p < 0.01 versus control group.

To experimentally validate the antitumor activities of predicted drugs, as well as their possible regulatory effects on RNA-MRs, we tested 5 of the 7 overlapped drugs in CMap and L1000FWD. Treatments with drugs for 24h inhibited cellular viability of SH-SY5Y cells in a dose-dependent manner (Figure 8A–8E). The IC50 were 1.26×10^-4^, 2.65×10^-7^, 6.39×10^-6^, 9.13×10^-7^, 1.92×10^-6^ mol/L for purvalanol A, idarubicin, WZ3146, AZD-8055, TG-101348, respectively. These drugs induced decreased expressions of ALYREF, ADAT1, ADAT3, and NSUN2. They also induced increased expressions of ALKBH5 and TET2 (Figure 8F). The validated 5 drugs had no previous report on regulation of RNA-MRs. Here the method was a relatively high accuracy model to predict inhibitors for RNA-MRs.

## DISCUSSION

Recently RNA-MRs emerged as important regulator in cancer [8]. In this study, it was found that the core prognostic gene set, consisted of RNA-MRs and their function-related genes, characterized NBL severity signature. They were rationally selected as potential drug target set for NBL. Then a promising ‘omics proximity strategy’ was used to analyze potential repositioning drugs for the target set. Inhibitors of HDAC, topoisomerase and CDK blocked upstream effectors of RNA modification, while mTOR and PI3K inhibitors blocked downstream effectors. HDAC inhibitors shared distinct drug-perturbed proteomic profiles with other kinds of inhibitors, which made us to conclude drug combination as an effective treatment. Finally, 5 out of 7 predicted drugs were experimentally confirmed. They had significantly inhibitory effects on neuroblastoma cells and regulated gene expressions of prognostic RNA-MRs.

The definition of drug target was important for linking drug response to genetic variation, understanding stratified clinical efficacy and safety, and predicting drug utility in patient subgroups [34]. Traditionally drugs were designed to target a single biological entity, usually a protein (the so-called “on-target”), with high selectivity to avoid mis-targeting (“off-targets”) [35]. However, the complexity of causative factors made such single-target drugs inadequate to achieve a therapeutic effect. Further, oncology was a therapeutic area which needed a substantial proportion of drug discovery efforts on the rational selection of mechanistic cancer drivers to be targeted [36]. Many of cancer drivers were newly discovered cancer-associated genes with little historical biological investigation and thus need a lot of time to yield useful targets for drug discovery [34]. Therefore, strategy for target identification and drug prediction here gave consideration for above issues, which also constituted the success factors of the strategy here. First, the prognostic gene set was identified based on transcriptome of NBL patients. The gene set was a rational reduced representation of DEGs, genes of which had closely functional relationship and co-expression patterns. Thus, the strategy here shortened the identification time of a novel clinically validated target set. Secondly, omics proximity strategy was used to predict drugs with reversed gene expressions of target set, which facilitated discovery of multi-target drugs. More importantly, we not only developed computational models to quantitatively identify potential targets and drugs, but also implemented experimental validations. Furthermore, the strategy here had a reliable and robust performance in all the validation schemas, thus it could be effectively applied to drugs prediction for other disease.

However, some limitations of this study should be mentioned. First, deep sequencing technologies would rapidly become cheaper while providing more accuracy and comprehensiveness. The performance of target identification method here could be further improved by more accuracy transcriptome data. Secondly, a more reliable measure of drug similarity would improve drug prediction. To do this, more omics data, including genome, transcriptome, proteomics and metabolomics, should be integrated to measure drug similarity. Besides, miRNAs regulated RNA-MRs and were possible targets for compounds. Computed models like HSSMMA or SNMFSMMA [37, 38] could be integrated to explain potential MOA for predicted drugs. Thirdly, all computational and experimental data were based on *in vitro* study, *in vivo* study could give more comprehensive data for further clinical trials. Finally, MOA of potential drug combinations and their experimental confirmation could be considered for the integrity of drug prediction. In future work, we will develop new tools and methods to overcome the current limitations.

RNA modification had important effects on many neuropsychiatric disorders [12]. The overexpressed m6A modification was a prognostic indicator for glioblastoma [39]. Genetic defects in m5C were linked to intellectual disability via regulating tRNA [40]. Here overexpressed m6A and m5C regulators were also closely associated with the malignancy of NBL, which confirmed their important role in tumor of peripheral nerve. RNA modification often occurred on cell-state-specific key regulatory transcripts for the gene-region-specific modifications [41]. Recent studies showed m6A modification regulated most aspects of RNA processing [8], and was regulated by miRNAs in neural stem cells [42, 43]. The deposition of m5C to tRNA stabilized RNA secondary structure and affected translational fidelity [44]. Here in severe NBL with significantly dysregulated RNA-MRs in cluster1, DEGs were mainly enriched in genetic information process (i.e., cell cycle and DNA replication), and glycolysis for energy supply (mTOR and PI3K-AKT signaling) [45]. Therefore RNA-MRs probably regulated the above process. Considering prognostic RNA-MRs had little structure information to characterize the druggability sites, inhibitors of upstream or downstream effectors of RNA-MRs may be potential multi-targets drugs.

NBL was characterized by frequent CNVs, such as chromosome 17 gain [1]. High-risk NBL contained segmental chromosomal aberrations, while low-risk NBL often presented with whole chromosomal gains [3]. Here in cluster1, ALYREF (Reader) amplified frequently, whereas simultaneous gain of ALYREF and ALKBH5 (Earser) ranked first in cluster2. Meanwhile aberrant expression of prognostic RNA-MRs was highly associated with their CNVs. Therefore, inhibitors regulating DNA methylation, histone modifications and RNA-associated silencing were good choices to block RNA-MRs via upstream effectors and avoid “off-target” induced by CNVs.

The computational omics proximity by query omics-based drug repurposing database could identify multi-target drugs reversing the omics profile induced by a biological state of interest [16]. Here the core prognostic gene set reproduced most significant features of total DEGs, namely, 80% drug overlap in L1000FWD database. There were 157 potential drugs predicted in CMap database, and 88 merging drugs targeting 13 kinds of targets in CMap and L1000FWD. Results reflected the following findings: a) biological functions related to RNA-MRs regulated overall DEG profile, and thus core prognostic genes supported possibility to find drugs to reverse overall DEG profile; b) the core prognostic genes were potential therapeutic targets for NBL; c) topoisomerase and mTOR inhibitors were studied well. Here we successfully rediscovered these known drugs and targets without using any prior drug information. Therefore, our method could give convinced identification of drugs with high therapy potential. The predicted CDK, PI3K, and HDAC inhibitors were promising, though with limited previous report for NBL therapy.

Here inhibitors of HDAC shared distinct drug-perturbed proteomic profile with kinase inhibitors. This was in line with previous studies, that combination of CDK and HDAC inhibitor could synergistically reduce oncogene expression [1]. Drug combinations overcame drug resistance and off-target effects, increased treatment efficacy and decrease drug dosage to avoid toxicity [46]. In Figure 7D, topoisomerase and exportin targeted were related to PLK, CDK, and aurora kinase. ALYREF, NSUN2, and ADARB1 were their therapeutic targets. HDAC and CDKs were related to RNA polymerase, and ALYREF was their therapeutic targets. TET2 regulated JAK and FLT3 and further regulating PI3K and MAPK signaling. This confirmed the cross-talk between RNA modifications provided an exquisite level of functional control [8, 12]. Two or more targets inhibiting RNA modification network together were more potential strategy for NBL therapy.

To summarize, this study provides an unbiased identification of drugs that can target NBL. We first compile the core prognostic gene set of RNA-MRs and their function-related prognostic genes. This set characterizes NBL severity signature. Drugs are collected and analyzed, which alter the expression of this set. Potential drugs are mainly HDAC, mTOR, PI3K, topoisomerase, and CDK inhibitors. Inhibitors of HDAC, topoisomerase and CDK block upstream effectors of RNA modification, while mTOR and PI3K inhibitors block downstream effectors. On the basis of the literature research, some of the drugs identified here were effective in neuroblastoma cells or NBL patients. Our experimental data also confirm the regulation effects of predicting drugs on RNA-MRs. We hope the information presented in this study will guide research community to further test and identify inhibitors for NBL in humans.

## MATERIALS AND METHODS

### Evaluation of the role of RNA-MRs in NBL disease course

The mRNA expression data were calculated from HTSeq-FPKM release of RNA-seq data. The log scale was applied for analyzing. The interaction and correlation among RNA-MRs were firstly analyzed by STRING (http://www.string-db.org/) and spearman correlation analysis, respectively. Consensus clustering was a popular algorithm for unsupervised classing in cancer research [47]. RNA-seq sample was then clustered into two groups (cluster1 and cluster2) by analyzing expression of RNA-MRs with “ConsensusClusterPlus” (K-means, 50 iterations, resample rate of 80%, and Pearson correlation) for R v3.6.0.

The Kaplan–Meier method with a two-sided log-rank test was used to compare the overall survival of the patients in two groups. The transcriptome differences between two groups were analyzed using PCA analysis (prcomp function in R). The DEGs between groups were calculated using limma in R. The consensus expression of RNA-MRs and the distribution of clinicopathological features in two groups were shown by pheatmap in R.

### Evaluation of CNVs for RNA-MRs and its relationship with their expression profile

When genes are drug targets, their CNVs affected gene expression abundance and protein structure, thus altered drug-binding pocket of receptors, therapeutic efficacy and safety of drugs [21]. Here CNVs were identified using segmentation analysis and GISTIC algorithm. The CNV patterns for each patient, as well as corresponding clinicopathological features were shown by ComplexHeatmap in R. The chromosome structure, location of CNV were represented using RCircos in R. To evaluate effects of CNVs on expressions of RNA-MRs, the expression levels were compared using One-way ANOVA or t-tests among NBLs with different CNV patterns.

### Functional differences in two NBL clusters defined by RNA-MRs

To functionally annotate DEGs, GO for BP, MF, and CC, together with KEGG pathway analysis were performed using the clusterProfiler, org.Hs.eg.db genome-wide annotation, and the enrichplot package in R. The top ten items were visualized by ggplot2 package.

To investigate the functional difference of whole transcriptome between clusters, GSEA (Broad Institute) was performed with hallmark gene set (v7.0). Gene sets with nominal P-value <0.05, and FDR <0.25 were considered to be significantly enriched.

### Evaluation of prognostic RNA-MRs

**Figure 9:**
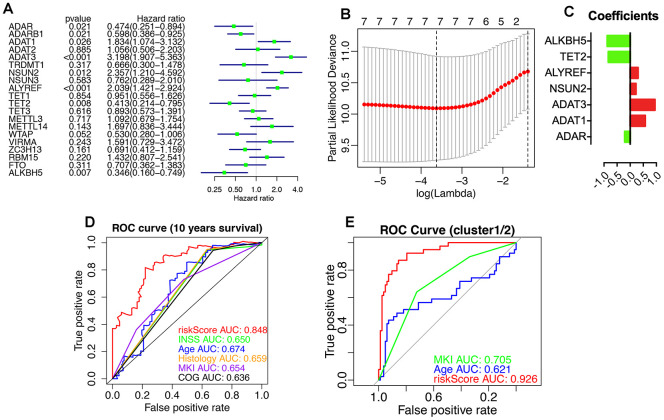
**Prognostic RNA modification regulators (RNA-MRs) and their prognostic values.** Prognostic RNA-MRs had a P<0.05 in univariate Cox regression analyses (**A**) and LASSO analysis (**B**, **C**). The riskScore calculated using prognostic RNA-MRs generated a convinced ROC curve (**D**, **E**).

The prognostic values of RNA-MRs were evaluated by univariate Cox regression and LASSO analyses (Figure 9). ADAR, ADARB1, ADAT1, ADAT3, NSUN2, ALYREF, TET2, and ALKBH5 were associated with survival significantly (P<0.05). Because L1000 high-throughput gene expression assay directly measured 978 genes and calculated related 12 000 genes instead of all transcriptome, there were only 10 RNA-MRs containing in CMap and L1000FWD. And 3 of them were prognostic regulators (ADAR, ADARB1, ADAT1), most of which were tumor suppressors and unsuitable for inhibitor design. Therefore, query by only RNA-MRs could not get convinced information retrieval of drugs targeting RNA-MRs, and this query result was used just as a reference.

### Preparation a core prognostic gene set for drug-perturbed expression profile analysis

Because the degree of co-expression was extremely high if genes were related to the same function [48], we then calculated co-expression correlation coefficient to obtain downstream or upstream related genes for prognostic RNA-MRs. Firstly, the association between overall survival and DEGs was evaluated by univariate Cox regression analyses, and 515 DEGs (P < 0.05) from total 2113 DEGs were considered as prognostic genes. Secondly, correlation between prognostic genes and prognostic RNA-MRs was evaluated by spearman correlation analysis (correlation coefficient >0.5 and p<0.001), and 213 genes from 515 DEGs were considered as function-related prognostic genes. Finally, core prognostic gene set consisted of 213 function-related prognostic genes and 8 prognostic RNA-MRs (Figure 5A).

### Transcriptome proximity analysis in L1000 FWD

To evaluate the importance of RNA-MRs and their downstream or upstream related genes, information retrieval of core prognostic gene set was analyzed as a reasonable reduced representation of total DEGs. The reversed expression profile of total 2113 DEGs and core prognostic gene set were used separately to query L1000 FWD (http://amp.pharm.mssm.edu/L1000FWD). The similarity scores of predicted drugs were given to quantify the similarity (i.e. overlap) between the input pair of up/down gene sets and the signature up/down genes in L1000FWD. Fisher exact test and subsequent Benjamini-Hochberg procedure were used to assess the statistical significance (*P* value). Then Z-score (z) was calculated for quantifying the deviation of the observed rank from the expected rank. The combined score (c) was calculated as “*c*=z·log10(P)”. Drugs with higher similarity scores (ranging from 0 to 1, *P*<0.05) were considered mimicking reversed expression profile of total DEGs or core prognostic gene set, and as potential therapeutically treatments.

### Transcriptome proximity analysis in CMap

The CMap (https://clue.io) was a large-scale compendium of functional perturbations (compounds, shRNAs, cDNAs, and biologics) coupled to an information-rich omics profile, which supplied a comprehensive analysis for perturbational class as well. However, there was a technical limit for input gene set (150 up/down-regulated genes). As a complementary of query in L1000FWD, the core prognostic gene set were used to query CMap.

The similarity of the query to each CMap signature was computed and yielded a rank-ordered list of the signatures. Three connectivity score metrics constituted a statistical framework to provide a holistic quantification of the similarity: (1) a nominal *P* value using the Kolmogorov-Smirnov enrichment statistic; (2) a FDR accounting for multiple hypothesis testing; (3) a connectivity score (tau) comparing reversed core prognostic gene set to perturbagen set (named “Touchstone”, TS) via weighted Kolmogorov-Smirnov enrichment statistical analysis. A tau>90 was used as convinced strong scores. To assess the universal targets of drugs, PCL describing compounds with the same mechanism of action (MOA) or biological functions was used to cluster perturbagens.

### Evaluation of merging drugs in CMap and L1000FWD

Firstly, structure and MOAs of drugs retrieved from both CMap and L1000FWD were analyzed. Then, structure and MOAs of drugs were analyzed when they shared same targets: 1) targets retrieved from both CMap and L1000FWD, or 2) drugs with similarity>0.3 in L1000FWD, or 3) PCLs with tau>99 or drug count>5 in CMap. A literature research for these drugs and targets were used to query their use in clinic or experiment for NBL.

### Proteomics proximity analysis in CMap

In CMap, two Proteomics Connectivity Hubs, P100 and GCP were queried to evaluate MOA similarities of potential drugs (tau>95). Drugs with different targets induced different protein change profiles, which represented different signaling pathways being affected. A similarity matrix of proteomic connectivity in available drugs was calculated by Pearson correlation.

### Network analysis for inhibitors of downstream or upstream effectors

The network analysis was used to analyze relationship between drug and targets, as well as between drug response biomarkers and prognostic RNA-MRs. All targets of drugs (tau>90 in CMap, or merging drugs in L1000FWD) were searched in STRING, and their basic interaction were downloaded. Interaction between function-related prognostic genes and RNA-MRs, and between RNA-MR themselves, were merged together using spearman correlation scores calculated above (score>0.5). Cytoscape was used to construct the network.

### Antitumor bioassay

### CCK8 assay

The human neuroblastoma cell line SH-SY5Y was cultured in RMPI 1640 medium containing 15% fetal bovine serum (FBS). Cells were seeded into 96-well plates (10 000 per well), treated for 24 h with different concentration of purvalanol A, idarubicin, WZ3146, AZD-8055, TG-101348 (10^-8^~10^-5^ mol/L, all purchased from Selleck, Shanghai, China). The cells were incubated at 37 °C for 2 h with CCK-8 Kit (Dojindo, Tokyo, Japan). Medium without cells was incubated with CCK8 as blank well. The optical densities (ODs) were checked at 450 nm using a microplate reader. The inhibition rate was calculated as following: (ODdrug-ODblank)/(ODcontrol-ODblank) × 100%.

### Quantitative real-time PCR

The mRNA expression of AlyREF, ADAT1, ADAT3, NSUN2, ALKBH5, TET2 and GAPDH were detected with qRT-PCR. Cells were seeded into 6-well plates (300 000 per well), treated with purvalanol A, idarubicin, WZ3146, AZD-8055, TG-101348 (5×10^-6^, 7×10^-8^, 5×10^-6^, 3×10^-7^, and 5×10^-6^ mol/L) for 24 h. The total RNA was extracted with TRIzol reagent (Invitrogen, Carlsbad, CA, USA) and the cDNA was reverse transcribed using ReverTra Ace (Toyobo, Osaka, Japan). The data were detected using SYBR Green PCR Master Mix (Toyobo) by ABI 7500 Fast (Applied Biosystems). Relative quantification of mRNA ratio of the target gene to GAPDH was calculated using the 2^−ΔΔCt^ method. The primers were in Table 3.

**Table 3:** Primers sequences used for Quantitative real-time PCR (Human).

**Gene**	**Primer sequence**
GAPDH	forward: 5′-CAAGGCTGTGGGCAAGGTCATC-3′
	reverse: 5 ′-GTGTCGCTGTTGAAGTCAGAGGAG-3′
AlyREF	forward: 5′-CCGACAAGTGGCAGCACGATC-3′
	reverse: 5 ′-GCCTCCACCACCACCAAAACC-3′
ADAT1	forward: 5′-CCAATCTCACCAGGCATCCACAG-3′
	reverse: 5 ′-GGCACTTCCCAATGACCACAGC-3′
ADAT3	forward: 5′-CGCCCTGGAGATGCTGCTTTG-3′
	reverse: 5 ′-CGCTGGTCACCTGCTTGTCC-3′
NSUN2	forward: 5′-GCAGAGACCAGAGAAAGCACACAG-3′
	reverse: 5 ′-TCAGCAGCACATTCCGCAACTC-3′
ALKBH5	forward: 5′-CGTGTCCGTGTCCTTCTTTAGCG-3′
	reverse: 5 ′-CTGACAGGCGATCTGAAGCATAGC-3′
TET2	forward: 5′-CGTGGATGAGTTTGGGAGTGTGG-3′
	reverse: 5 ′-GCTGTGGTGGCTGCTTCTGTAG-3′

### Statistical analysis

The chi-square tests were used to compare the distribution of gender, age, INSS, MYCN, ploidy, histology, grade, MKI, and COG risks.

The association between expression of RNA-MRs and clinicopathological characteristics, as well as CNVs and clinicopathological characteristics, were analyzed with chi-square test or Mann-Whitney U test.

All statistical analyses and figures were conducted using R v3.6.0 (https://www.r-project.org/), SPSS 20.0 (IBM, Chicago, USA) and GraphPad Prism 6.0 (GraphPad Software, La Jolla, CA, USA). All statistical results with a P <0.05 were considered to be significant.

### Ethics statement and dataset collection

Three datasets, clinical dataset, RNA-seq dataset, and CNV dataset were retrieved from TARGET and TCGA databases (Supplementary Figure 1D). The data was accessed according to open access guidelines where written informed consents were obtained in accordance with the local institutional review boards. Clinicopathological information was summarized in Table 1. To evaluate the information retrieval of patients with RNA-seq and CNV data for overall NBL patients, univariate and multivariate Cox regression analyses were performed, and the association between clinicopathological factors and overall survival of patients in 3 datasets was determined.

## Supplementary Material

Supplementary Figures

Supplementary Tables
