# Supplementary material for: Target RNA modification for epigenetic drug repositioning in neuroblastoma: computational omics proximity between repurposing drug and disease
Source: Aging (Albany NY). 2020 Oct 12;12(19):19022–44. doi: 10.18632/aging.103671 (PMC7732279; doi:10.18632/aging.103671)
Supplement: Supplementary Figures [file aging-12-103671-s001..pdf]

SUPPLEMENTARY FIGURES

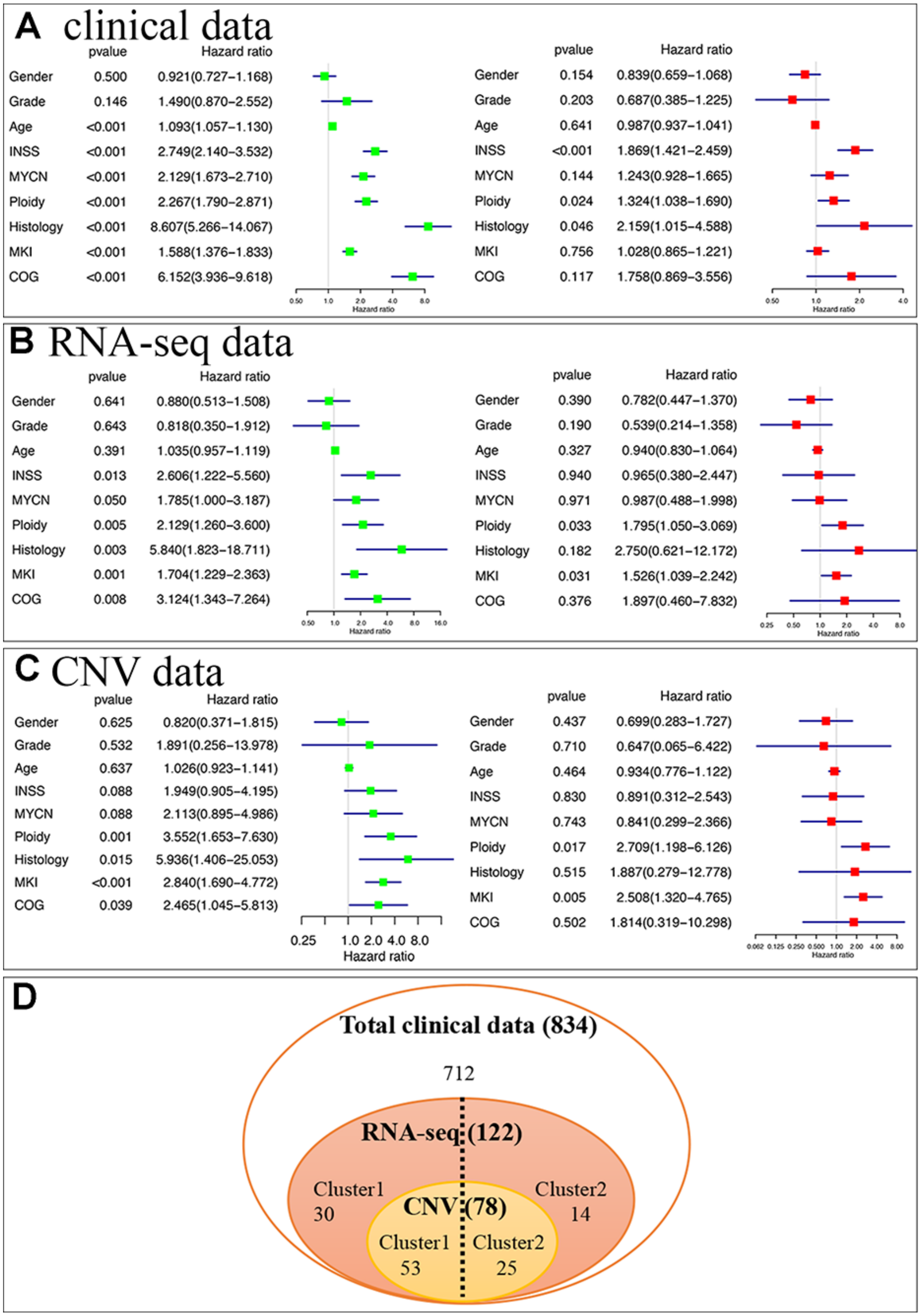

Supplementary Figure 1. Univariate (left) and multivariate Cox (right) regression analyses for the association between clinicopathology and overall survival in clinical sample (**A**), RNA-seq sample (**B**), and CNV sample (**C**) datasets. (**D**) Relationship among three datasets.

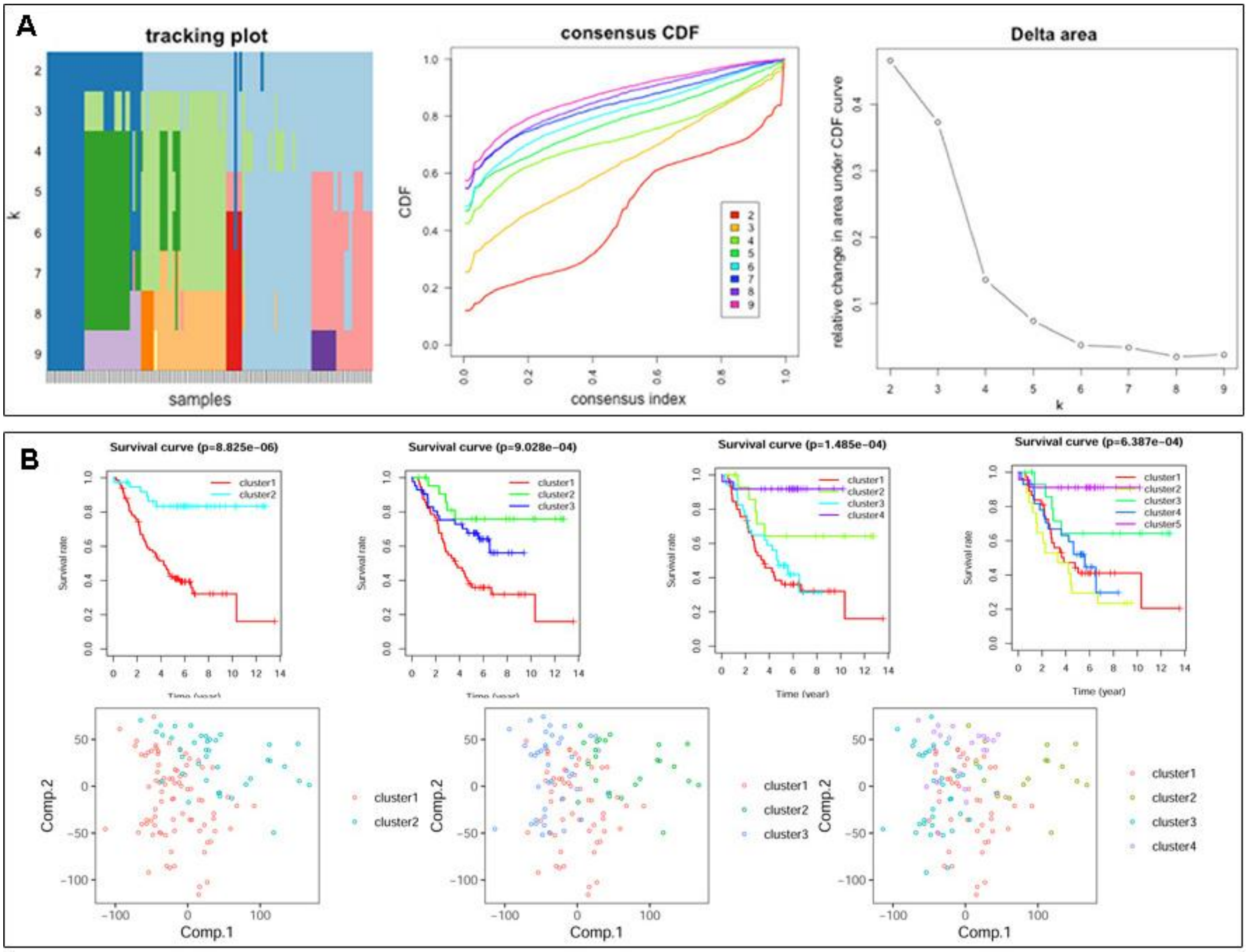

Supplementary Figure 2. Identification of consensus clusters by RNA modification regulators (RNA-MRs). (**A**) The tracking plot, cumulative distribution function (CDF), relative change in area under CDF curve (delta area) for k=2 to k=10. (**B**) Kaplan–Meier overall survival curves, and principal component analysis for k=2 to k=5.

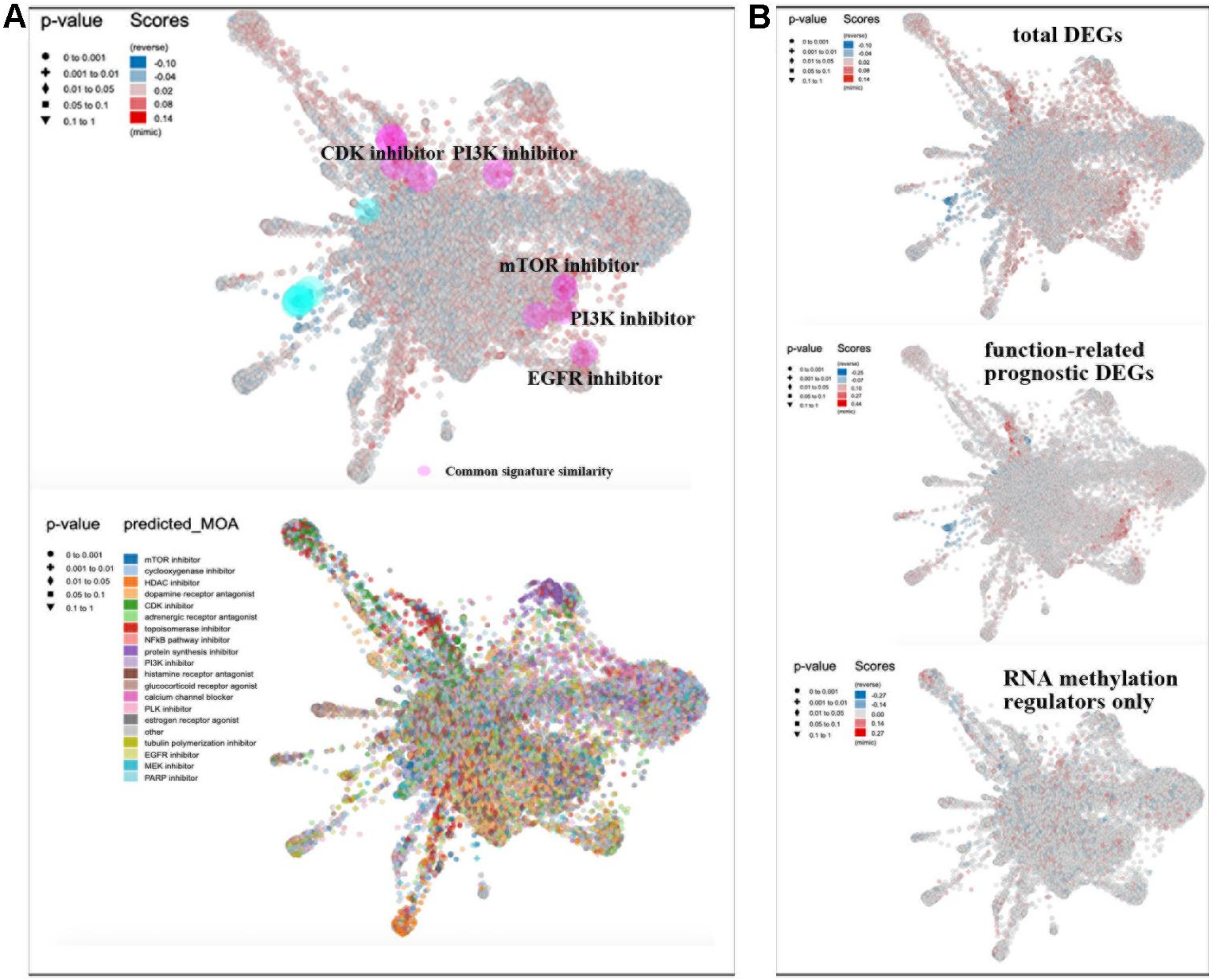

Supplementary Figure 3. L1000FWD visualization of the signature similarity mapped to all reference drugs. (**A**) L1000FWD visualization of overlap drugs with reversed expression profile of total DEGs and function-related prognostic DEGs. Pink ring in map represented the most convinced drugs overlapped in two queries. (**B**) Drug-perturbed expression profiles similar to the reversed profiles of total DEGs, core prognostic gene set, and RNA modification regulators. Score scale (blue to red) represents opposite to similar results.
