# Supplementary material for: Target RNA modification for epigenetic drug repositioning in neuroblastoma: computational omics proximity between repurposing drug and disease
Source: Aging (Albany NY). 2020 Oct 12;12(19):19022–44. doi: 10.18632/aging.103671 (PMC7732279; doi:10.18632/aging.103671)
Supplement: Supplementary Tables [file aging-12-103671-s002..pdf]

Table S1. The top 50 drugs with opposed expression profiles of total DEGs in L1000FWD.

Drug	Similarity score	Padj-value	Combined score	Cell	Time	InChIKey	Molecular formula	Molecular wt
naproxol	0.1407	3.35E-73	131.76	MCF7	24H	LTRANDSQVZFZDG-SNVBAGLBSA-N	C14H16O2	216.276
palbociclib	0.1363	2.52E-67	125.91	BT20	24H	AHJRHEGDXFFMBM-UHFFFAOYSA-N	C24H29N7O2	447.533
BRD-K68548958	0.1354	1.05E-68	116.85	HCC515	24H	HEKJYZZSCQBJGB-XDHOZWIPSA-N	C24H19N3O6	445.424
amsacrine	0.131	2.92E-64	116.52	MCF7	24H	JRNUZHHDXNSOMO-UHFFFAOYSA-N	C22H20N2O3S	392.471
ingenol	0.1266	1.11E-58	98.94	HCC515	24H	GIMKEHNOTHXONN-UHFFFAOYSA-N	C34H36O7	556.645
SIB-1893	0.1223	1.1E-57	105.94	HEPG2	24H	SISOFUCTXZKSOQ-UHFFFAOYSA-N	C14H13N	195.26
palbociclib	0.1205	1.4E-54	101.29	BT20	24H	AHJRHEGDXFFMBM-UHFFFAOYSA-N	C24H29N7O2	447.533
homosalate	0.1187	4.67E-53	95.59	MCF7	24H	WSSJONWNBBTCMG-UHFFFAOYSA-N	C16H22O3	262.344
palbociclib	0.1161	8.04E-57	109.36	MDAMB231	24H	AHJRHEGDXFFMBM-UHFFFAOYSA-N	C24H29N7O2	447.533
ZK-164015	0.1161	6.96E-50	91.19	MCF7	24H	LYJSJVYJLZOMCD-UHFFFAOYSA-N	C30H43NO4S	513.732
BRD-K65955264	0.1152	3.97E-52	93.65	MCF7	24H	OQPXCZGFVBKXJK-UHFFFAOYSA-N	C21H29N2	437.381
devazepide	0.1152	8.74E-49	86.8	MCF7	24H	NFHRQQKPEBFUJK-HSZRJFAPSA-N	C25H20N4O2	408.452
diphenyleneiodonium	0.1152	1.08E-49	90.45	MCF7	24H	QFXKXRXFBRLLPQ-UHFFFAOYSA-N	C12H8I	279.096
teniposide	0.1126	2.57E-49	90.95	MCF7	24H	NRUKOCRGYNPUPR-OQMCATNJSA-N	C32H32O13S	656.654
BRD-K30836161	0.1099	1.49E-47	82.72	A375	24H	NRYSIYZIRHQJSQ-UHFFFAOYSA-N	C16H13F2N3OS2	479.444
SIB-1893	0.1099	1.05E-47	87.63	MCF7	24H	SISOFUCTXZKSOQ-UHFFFAOYSA-N	C14H13N	195.26
spironolactone	0.1099	1.58E-44	73.17	VCAP	24H	LXMSZDCAJNLERA-ZHYRCANASA-N	C24H32O4S	416.573
etoposide	0.1091	6.87E-48	82.58	A549	24H	SYKHAYBRYKMZLP-LTGYLHEKSA-N	C29H32O13	588.557
palbociclib	0.1091	6.96E-50	96.49	MDAMB231	24H	AHJRHEGDXFFMBM-UHFFFAOYSA-N	C24H29N7O2	447.533
niguldipine	0.1064	8.17E-42	75.25	MCF7	24H	SVJMLYUFVDMUHP-UHFFFAOYSA-N	C36H39N3O6	609.711
BRD-K29506255	0.1055	1.07E-44	78.59	A375	24H	IMPABEWSEMMGHP-UHFFFAOYSA-N	C16H14N2O2S2	444.448
FCCP	0.1055	3.09E-44	74.88	PC3	24H	BMZRVOVNUMQTIN-UHFFFAOYSA-N	C10H5F3N4O	254.168
emodic-acid	0.1047	4.78E-43	78.45	MCF7	24H	ZJXVNNSMRGTDBI-UHFFFAOYSA-N	C15H8O7	300.22
BRD-K00313977	0.1038	3.53E-43	76.27	MCF7	24H	CZIACNCULOWAAI-UHFFFAOYSA-N	C18H19N3OS2	471.516
tremulacin	0.1038	4.78E-43	76.31	MCF7	24H	RCKCYCDBDYUIGM-LFMHJWGUSA-N	C27H28O11	528.505
Y-134	0.1038	2.76E-42	77.32	MCF7	24H	LQEOPHGPHCWOAC-UHFFFAOYSA-N	C28H28N2O3S	472.599
aminopurvalanol-a	0.1011	8.86E-42	75.93	MCF7	24H	RAMROQQYRRQPDL-HNNXBMFYSA-N	C19H26ClN7O	403.909

FCCP	0.1011	4.68E-40	73.97	MCF7	24H	BMZRVOVNUMQTIN-UHFFFAOYSA-N	C10H5F3N4O	254.168
palbociclib	0.1011	1.06E-37	69.58	BT20	24H	AHJRHEGDXFFMBM-UHFFFAOYSA-N	C24H29N7O2	447.533
idarubicin	0.1003	5.3E-38	69.97	A549	24H	XDXDZDZNSLXDNA-YMYDAGKFSA-N	C26H27NO9	497.494
aminopurvalanol-a	0.0994	1.22E-40	70.01	PC3	24H	RAMROQQYRRQPDL-HNNXBMFYSA-N	C19H26ClN7O	403.909
BRD-K38625260	0.0994	1.11E-36	62.85	A549	24H	VPTSFXDCXJGRBR-UHFFFAOYSA-N	C19H19F2N3OS2	521.524
7b-cis	0.0985	1.37E-35	59.51	MCF7	24H	AICKZRPQDLFSPA-SREVYHEPSA-N	C12H11ClN4O2	278.694
cytochalasin-b	0.0976	2.89E-36	61.06	A375	24H	GBOGMAARMMDZGR-JNQYJPGFSA-N	C29H37NO5	479.608
teniposide	0.0976	2.29E-37	64.26	MCF7	24H	NRUKOCRGYNPUPR-OQMCATNJSA-N	C32H32O13S	656.654
BRD-K78385490	0.0967	6.57E-38	68.18	A549	24H	BPJXTVCIZAREGR-UHFFFAOYSA-N	C15H12N2O	350.292
gemcitabine	0.0967	1.26E-37	70.04	MCF7	24H	SDUQYLNIPVEERB-QPPQHZFASA-N	C9H11F2N3O4	263.198
palbociclib	0.0967	3.93E-39	72.1	MCF7	24H	AHJRHEGDXFFMBM-UHFFFAOYSA-N	C24H29N7O2	447.533
phorbol-12-myristate-13-acetate	0.0967	6.59E-35	59.03	HCC515	24H	PHEDXBVPIONUQT-UHFFFAOYSA-N	C36H56O8	616.825
rhodomyrtoxin-b	0.0959	3.95E-36	61.38	HT29	24H	SGFCERQKVVMREN-UHFFFAOYSA-N	C24H28O7	428.475
WZ-3146	0.095	9.31E-35	62.34	MCF7	24H	APHGZZPEOCCYNO-UHFFFAOYSA-N	C24H25ClN6O2	464.947
danazol	0.0941	4.75E-34	57.17	VCAP	24H	POZRVZJJTULAOH-LHZXLZLDSA-N	C22H27NO2	337.455
fulvestrant	0.0941	5.87E-33	60.18	MCF7	24H	VWUXBMIQPBEWFH-UHFFFAOYSA-N	C32H47F5O3S	606.771
BRD-K18726304	0.0923	3.26E-32	55.54	MCF7	24H	VYROYTKFGTZBKQ-UHFFFAOYSA-N	C22H23N3OS2	523.591
norethisterone	0.0923	1.83E-32	53.97	VCAP	24H	VIKNJXKGJWUCNN-XGXHKTLJSA-N	C20H26O2	298.419
BRD-K38625260	0.0915	3.48E-33	57.43	MCF7	24H	VPTSFXDCXJGRBR-UHFFFAOYSA-N	C19H19F2N3OS2	521.524
SA-1017940	0.0906	8.58E-35	66.26	PC3	6H	ZBLCOVKAEUNWGN-LWPQGVMLSA-N	C33H43N5O4	573.726
BRD-K50204028	0.0897	1.48E-30	56.31	VCAP	24H	UHIMEEWAODCTDO-XRMYXDSJSA-N	C39H47N5O6	681.82
tanespimycin	0.0897	1.48E-30	56.05	VCAP	48H	AYUNIORJHRXIBJ-ZGQRYRSUSA-N	C31H43N3O8	585.688
wortmannin	0.0897	5.3E-34	58.09	VCAP	24H	QDLHCMPXEPAAMD-ZGSWIPFCSA-N	C23H24O8	428.432

Table S2. The top 50 drugs with opposed expression profiles of core prognostic gene set in L1000FWD.

Drug	Similarity score	Padj-value	Combined score	Cell	Time	InChIKey	Molecular formula	Molecular wt
palbociclib	0.4825	1.45E-70	132.87	BT20	24H	AHJRHEGDXFFMBM-UHFFFAOYSA-N	C24H29N7O2	447.533
naproxol	0.4685	1.12E-67	124.15	MCF7	24H	LTRANDSQVZFZDG-SNVBAGLBSA-N	C14H16O2	216.276
palbociclib	0.4615	3.94E-68	123.96	BT20	24H	AHJRHEGDXFFMBM-UHFFFAOYSA-N	C24H29N7O2	447.533
amsacrine	0.4406	9.03E-63	113.94	MCF7	24H	JRNUZHHDXNSOMO-UHFFFAOYSA-N	C22H20N2O3S	392.471
BRD-K65955264	0.4126	1.68E-57	103.3	MCF7	24H	OQPXCZGFVBKXJK-UHFFFAOYSA-N	C21H29N2	437.381
diphenyleneiodonium	0.4056	9.79E-55	99.55	MCF7	24H	QFXKXRXFBRLLPQ-UHFFFAOYSA-N	C12H8I	279.096
palbociclib	0.3986	6.54E-56	109.28	MDAMB231	24H	AHJRHEGDXFFMBM-UHFFFAOYSA-N	C24H29N7O2	447.533
BRD-K68548958	0.3916	1.65E-54	95.43	MCF7	24H	HEKJYZZSCQBJGB-XDHOZWIPSA-N	C24H19N3O6	445.424
ingenol	0.3916	8.26E-52	87.27	HCC515	24H	GIMKEHNOTHXONN-UHFFFAOYSA-N	C34H36O7	556.645
BRD-K29506255	0.3776	1.36E-50	88.81	A375	24H	IMPABEWSEMMGHP-UHFFFAOYSA-N	C16H14N2O2S2	444.448
idarubicin	0.3776	5.13E-49	90.08	A549	24H	XDXDZDZNSLXDNA-YMYDAGKFSA-N	C26H27NO9	497.494
7b-cis	0.3706	4.57E-47	78.34	MCF7	24H	AICKZRPQDLFSPA-SREVYHEPSA-N	C12H11ClN4O2	278.694
malonoben	0.3706	2.01E-46	81.73	PC3	24H	MKUVGSJPVXTADZ-UHFFFAOYSA-N	C18H22N2O	282.38
ZK-164015	0.3642	2.98E-52	96.87	MCF7	24H	LYJSJVYJLZOMCD-UHFFFAOYSA-N	C30H43NO4S	513.732
aminopurvalanol-a	0.3636	3.28E-48	82.92	PC3	24H	RAMROQQYRRQPDL-HNNXBMFYSA-N	C19H26ClN7O	403.909
BRD-K00313977	0.3636	9.13E-48	84.18	MCF7	24H	CZIACNCULOWAAI-UHFFFAOYSA-N	C18H19N3OS2	471.516
FCCP	0.3636	3.83E-47	86.84	MCF7	24H	BMZRVOVNUMQTIN-UHFFFAOYSA-N	C10H5F3N4O	254.168
BRD-K18726304	0.3566	5.13E-45	77.06	MCF7	24H	VYROYTKFGTZBKQ-UHFFFAOYSA-N	C22H23N3OS2	523.591
danazol	0.3566	2.43E-45	75.73	VCAP	24H	POZRVZJJTULAOH-LHZXLZLDSA-N	C22H27NO2	337.455
homosalate	0.3566	2.06E-45	81.64	MCF7	24H	WSSJONWNBBTCMG-UHFFFAOYSA-N	C16H22O3	262.344
mestanolone	0.3566	2.31E-45	80.15	VCAP	24H	WYZDXEKUWRCKOB-YDSAWKJFSA-N	C20H32O2	304.467
norethisterone	0.3566	2.35E-43	74.56	VCAP	24H	VIKNJXKGJWUCNN-XGXHKTLJSA-N	C20H26O2	298.419
oxymetholone	0.3566	2.07E-43	78.67	VCAP	24H	DLZUBMGMHUNDLF-CVSXEEBHSA-N	C21H32O3	332.477
SIB-1893	0.3519	5E-51	97.51	MCF7	24H	SISOFUCTXZKSOQ-UHFFFAOYSA-N	C14H13N	195.26
spironolactone	0.3519	3.34E-49	82.01	VCAP	24H	LXMSZDCAJNLERA-ZHYRCANASA-N	C24H32O4S	416.573
AG-879	0.3497	5.13E-44	80.11	MCF7	24H	XRZYELWZLNAXGE-KPKJPENVSA-N	C18H24N2OS	316.461
fulvestrant	0.3497	2.18E-43	78.69	MCF7	24H	VWUXBMIQPBEWFH-UHFFFAOYSA-N	C32H47F5O3S	606.771

niguldipine	0.3497	2.6E-40	77.61	MCF7	24H	SVJMLYUFVDMUHP-UHFFFAOYSA-N	C36H39N3O6	609.711
palbociclib	0.3497	3.3E-45	84.06	MCF7	24H	AHJRHEGDXFFMBM-UHFFFAOYSA-N	C24H29N7O2	447.533
palbociclib	0.3497	2.35E-43	79.35	BT20	24H	AHJRHEGDXFFMBM-UHFFFAOYSA-N	C24H29N7O2	447.533
tanespimycin	0.3457	2.16E-48	92.06	VCAP	48H	AYUNIORJHRXIBJ-ZGQRYRSUSA-N	C31H43N3O8	585.688
BRD-K30836161	0.3427	1.7E-43	75.38	A375	24H	NRYSIYZIRHQJSQ-UHFFFAOYSA-N	C16H13F2N3OS2	479.444
BRD-K38625260	0.3427	5.07E-42	71.62	A549	24H	VPTSFXDCXJGRBR-UHFFFAOYSA-N	C19H19F2N3OS2	521.524
FCCP	0.3427	1.44E-43	73.44	PC3	24H	BMZRVOVNUMQTIN-UHFFFAOYSA-N	C10H5F3N4O	254.168
PI-828	0.3395	7.08E-48	81.76	VCAP	24H	WUKMIBOGGXMBAC-UHFFFAOYSA-N	C19H18N2O3	322.358
AZD-8055	0.3357	2.56E-42	73.47	VCAP	24H	KVLFRAWTRWDEDF-IRXDYDNUSA-N	C25H31N5O4	465.545
devazepide	0.3357	8.43E-41	72.31	MCF7	24H	NFHRQQKPEBFUJK-HSZRJFAPSA-N	C25H20N4O2	408.452
emodic-acid	0.3357	5.6E-42	76.22	MCF7	24H	ZJXVNNSMRGTDBI-UHFFFAOYSA-N	C15H8O7	300.22
palbociclib	0.3357	2.43E-45	84.04	MDAMB231	24H	AHJRHEGDXFFMBM-UHFFFAOYSA-N	C24H29N7O2	447.533
aminopurvalanol-a	0.3287	2.18E-41	74.9	MCF7	24H	RAMROQQYRRQPDL-HNNXBMFYSA-N	C19H26ClN7O	403.909
CGK-733	0.3287	1.64E-40	67.82	PC3	24H	HLCDNLNLQNYZTK-UHFFFAOYSA-N	C23H18Cl3FN4O3S	555.836
FCCP	0.3287	2.34E-39	65.13	HCC515	24H	BMZRVOVNUMQTIN-UHFFFAOYSA-N	C10H5F3N4O	254.168
rhodomyrtoxin-b	0.3272	2.94E-45	82.16	HT29	24H	SGFCERQKVVMREN-UHFFFAOYSA-N	C24H28O7	428.475
SIB-1893	0.321	2.12E-44	82.21	HEPG2	24H	SISOFUCTXZKSOQ-UHFFFAOYSA-N	C14H13N	195.26
tremulacin	0.321	1.45E-44	79.48	MCF7	24H	RCKCYCDBDYUIGM-LFMHJWGUSA-N	C27H28O11	528.505
PP-110	0.3148	9.97E-42	72.57	HT29	24H	ULPBTMCLMXCXEB-UHFFFAOYSA-N	C15H15N7	293.327
teniposide	0.3086	1.16E-41	82.08	MCF7	24H	NRUKOCRGYNPUPR-OQMCATNJSA-N	C32H32O13S	656.654
WZ-3146	0.3086	4.89E-41	76.22	MCF7	24H	APHGZZPEOCCYNO-UHFFFAOYSA-N	C24H25ClN6O2	464.947
wortmannin	0.3025	1.35E-41	71.08	VCAP	24H	QDLHCMPXEPAAMD-ZGSWIPFCSA-N	C23H24O8	428.432
Y-134	0.3025	2.49E-40	76.36	MCF7	24H	LQEOPHGPHCWOAC-UHFFFAOYSA-N	C28H28N2O3S	472.599

Table S3. The merging drugs with opposed expression profiles of total DEGs (T), core prognostic gene set (C), and RNA modification regulators (R) in L1000FWD (Top 50).

Drug	Similarity score			Combined score			MOA	Main Targets	Literature report
	T	C	R	T	C	R			
palbociclib	0.136	0.483		125.91	132.87		CDK inhibitor	CDK 4/6, CCND3	In vitro
naproxol	0.141	0.469		131.76	124.15		Anti-inflammatory	PTGS 1/2	
**amsacrine**	**0.131**	**0.441**	**0.182**	**116.52**	**113.94**	**3.06**	**Topoisomerase inhibitor**	**TOP2A, KCNH2**	**Clinic**
BRD-K65955264	0.115	0.413		93.65	103.3		Calcium channel blocker		
diphenyleneiodonium	0.115	0.406		90.45	99.55		NO synthase inhibitor	ALDH 1A2/2/5A1/7A1, NOX3, XDH	Clinic
SIB-1893	0.122	0.392		105.94	97.51		Glutamate receptor antagonist	GRM 5/4	
ZK-164015	0.116	0.399		91.19	96.87		Estrogen receptor antagonist	ESR 1/2	
tanespimycin	0.090	0.385		56.05	92.06		mTOR inhibitor		In vitro
**idarubicin**	**0.100**	**0.378**	**0.182**	**69.97**	**90.08**	**3.1**	**Topoisomerase inhibitor**	**TOP2A**	**Clinic**
BRD-K29506255	0.106	0.378		78.59	88.81		PI3K inhibitor		
**ingenol**	**0.127**	**0.392**	**0.182**	**98.94**	**87.27**	**3.24**	**PKC activator**	**PRK CD/CE**	**In vitro**
FCCP	0.106	0.364		74.88	86.84		MOP uncoupler		In vitro
BRD-K00313977	0.104	0.364		76.27	84.18		PI3K inhibitor		
aminopurvalanol-a	0.101	0.364		75.93	82.92		CDK inhibitor	CDK 1/2/5/6	
rhodomyrtoxin-b	0.096	0.371		61.38	82.16		SFU inhibitor		
teniposide	0.113	0.350		90.95	82.08		Topoisomerase inhibitor	TOP2A, CYP3A5	Clinic
spironolactone	0.110	0.385		73.17	82.01		MR antagonist	NR3 C2/C1, AR, CACN A1/A2/B1/B2/B3/B4/G1	
homosalate	0.119	0.357		95.59	81.64		HSP inducer		
tremulacin	0.104	0.350		76.31	79.48		Lipoxygenase inhibitor		
fulvestrant	0.094	0.350		60.18	78.69		Estrogen receptor antagonist	ESR 1/2, EPHX2, GPER1	
7b-cis	0.099	0.371		59.51	78.34		Exportin antagonist	XPO1	
niguldipine	0.106	0.350		75.25	77.61		Calcium channel blocker	ADRA1 A/B/D, CACNA1C	In vitro
BRD-K18726304	0.092	0.357		55.54	77.06		mTOR inhibitor		
Y-134	0.104	0.336		77.32	76.36		Estrogen receptor antagonist	ESR 1/2	
emodic-acid	0.105	0.336		78.45	76.22		Laxative		
WZ-3146	0.095	0.343		62.34	76.22		EGFR inhibitor	EGFR	

danazol	0.094	0.357	57.17	75.73	Estrogen receptor antagonist	ESR1, GNRH R/R2, AR, CCL2, CYP2C8, PGR, TNF	
BRD-K30836161	0.110	0.343	82.72	75.38	PI3K inhibitor		
norethisterone	0.092	0.357	53.97	74.56	Progesterone receptor agonist	PGR	
devazepide	0.115	0.336	86.8	72.31	CCK receptor antagonist	CCKAR, CCKBR, GAST	In vitro
BRD-K38625260	0.099	0.343	62.85	71.62	mTOR inhibitor		
wortmannin	0.090	0.329	58.09	71.08	PI3K inhibitor	PIK3 CA/CG/CD/R1, PLK 1/3, ATM, ATR, MTOR	In vitro

MOA: Mechanism of action; MOP: Mitochondrial oxidative phosphorylation; MR: Mineralocorticoid receptor; SFU: sodium fluorescein uptake

Table S4. Drugs with opposed expression profiles of core prognostic gene set in CMap (Score>90).

Rank	Score	Name	InChIKey	Molecular_formula	MOA	Main target
**14**	**99.79**	**palbociclib**	**AHJRHEGDXFFMBM-UHFFFAOYSA-N**	**C24H29N7O2**	**CDK inhibitor**	**CDK 4/6, CCND3**
**26**	**99.65**	**aminopurvalanol-a**	**RAMROQQYRRQPDL-HNNXBMFYSA-N**	**C19H26ClN7O**	**CDK inhibitor**	**CDK 1/2/5/6**
33	99.54	HSP90-inhibitor	OWPMENVYXDJDOW-UHFFFAOYSA-N	C20H20N2O4	HSP inhibitor	HSP90 AA1/AB1
56	99.26	purvalanol-a	PMXCMJLOPOFPBT-HNNXBMFYSA-N	C19H25ClN6O	CDK/DYRK inhibitor	CDK1/2/4/5, CCN D1/E1, CSNK1G3
60	99.26	selumetinib	CYOHGALHFOKKQC-UHFFFAOYSA-N	C17H15BrClFN4O3	MEK inhibitor	MAP2K 1/2
72	99.05	WYE-125132	QLHHRYZMBGPBJG-UHFFFAOYSA-N	C27H33N7O4	MTOR inhibitor	MTOR, PIK3CA
78	98.94	crizotinib	KTEIFNKAUNYNJU-GFCCVEGCSA-N	C21H22Cl2FN5O	ALK inhibitor	ALK, MET, CYP 2B6/3A5
79	98.94	WYE-354	IMXHGCRIEAKIBU-UHFFFAOYSA-N	C24H29N7O5	MTOR inhibitor	MTOR
81	98.91	AZD-8055	KVLFRAWTRWDEDF-IRXDYDNUSA-N	C25H31N5O4	MTOR inhibitor	MTOR
83	98.87	PI-103	TUVCWJQQGGETHL-UHFFFAOYSA-N	C19H16N4O3	MTOR/PI3K inhibitor	PIK3 CA/CG/CB/CD, MTOR, PRKDC
84	98.87	ISOX	WWGBHDIHIVGYLZ-UHFFFAOYSA-N	C22H30N4O6	HDAC inhibitor	HDAC6
89	98.84	KU-0060648	AATCBLYHOUOCTO-UHFFFAOYSA-N	C33H34N4O4S	DNA-PK/PI3K inhibitor	PIK3 CA/CB/CD/CG, PRKDC
92	98.8	dactolisib	JOGKUKXHTYWRGZ-UHFFFAOYSA-N	C30H23N5O	MTOR/PI3K/PK inhibitor	MTOR, PIK3CA/CG/CD/CB, ATR
**95**	**98.73**	**WZ-3146**	**APHGZZPEOCCYNO-UHFFFAOYSA-N**	**C24H25ClN6O2**	**EGFR inhibitor**	**EGFR**
96	98.73	TG-101348	JOOXLOJCABQBSG-UHFFFAOYSA-N	C27H36N6O3S	FLT3/JAK inhibitor	JAK 2/1/3, FLT3, BRD4
99	98.7	BMS-754807	LQVXSNNAFNGRAH-QHCPKHFHSA-N	C23H24FN9O	IGF-1 inhibitor	IGF1R, AKT1
103	98.63	vorinostat	WAEXFXRVDQXREF-UHFFFAOYSA-N	C14H20N2O3	HDAC inhibitor	HDAC 1/2/3/6/8/10/11/5/9
109	98.56	NCH-51	MDYDGUOQFUQOGE-UHFFFAOYSA-N	C20H26N2O2S2	HDAC inhibitor	HDAC 1/10/11/2/C3/4/5/6/7/8/9
110	98.52	KU-0063794	RFSMUFRPPYDYRD-CALCHBBNSA-N	C25H31N5O4	MTOR inhibitor	MTOR
112	98.48	GDC-0941	LHNIIDJUOCFXAP-UHFFFAOYSA-N	C23H27N7O3S2	PI3K inhibitor	PIK3 CG/CA/CB/CD
116	98.45	BMS-536924	FDBVBFZVZLTNFJ-KKPVDXMRSA-N	C25H26ClN5O3	IGF-1 inhibitor	IGF1R, AKT1, CCNE1
119	98.41	NVP-TAE684	QQWUGDVOUVUTOY-UHFFFAOYSA-N	C30H40ClN7O3S	ALK inhibitor	ALK, INSR
122	98.38	APHA-c8	UFQOXIMRSMFQRI-BQYQJAHWSA-N	C16H16N2O3	HDAC inhibitor	HDAC8
123	98.38	PI-828	WUKMIBOGGXMBAC-UHFFFAOYSA-N	C19H18N2O3	PI3K inhibitor	
128	98.34	AS-605240	SQWZFLMPDUSYGV-UXBLZVDNSA-N	C12H7N3O2S	PI3K inhibitor	MAOB, PIK3 CA/CB/CD/CG
**131**	**98.31**	**7b-cis**	**AICKZRPQDLFSPA-SREVYHEPSA-N**	**C12H11ClN4O2**	**Exportin antagonist**	**XPO1**
138	98.2	PF-562271	MZDKLVOWGIOKTN-UHFFFAOYSA-N	C21H20F3N7O3S	FAK inhibitor	PTK 2/2B
140	98.17	dactinomycin	QKRYGAZNLBVXHK-UHFFFAOYSA-N	C62H86N12O16	RNAP inhibitor	POLR2A

147	98.11	entinostat	INVTYAOGFAGBOE-UHFFFAOYSA-N	C21H20N4O3	HDAC inhibitor	HDAC1/2/3/9
148	98.1	CGP-60474	IYNDTACKOAXKBJ-UHFFFAOYSA-N	C18H18ClN5O	CDK inhibitor	CDK1/2
150	98.06	staurosporine	HKSZLNNOFSGOKW-FYTWVXJKSA-N	C28H26N4O3	PKC inhibitor	CDK 2/1/5, GSK3B, CAMK2B
151	98.06	chromomycin-a3	LNGHEQSPMNWBCU-RUGPHKIXSA-N	C56H82O24	DNA binding agent	
152	98.06	AS-601245	RCYPVQCPYKNSTG-UHFFFAOYSA-N	C20H16N6S	JNK inhibitor	GSK3B, MAPK10/8/9, PIM1
153	98.05	simvastatin	RYMZZMVNJRMUDD-UHFFFAOYSA-N	C25H38O5	HMGCR inhibitor	HMGCR, CYP 2C8/3A4/3A5, ITGB2
155	98.03	PHA-793887	HUXYBQXJVXOMKX-UHFFFAOYSA-N	C19H31N5O2	CDK inhibitor	CDK1/2/4/5/7/9, CCN D1/E1
159	98	pidorubicine	AOJJSUZBOXZQNB-VTZDEGQISA-N	C27H29NO11	Topoisomerase inhibitor	TOP2A
160	97.99	WT-171	KXWWYFKVBFUVIZ-SGWCAAJKSA-N	C27H30N4O3	HDAC inhibitor	HDAC6
162	97.98	geldanamycin	QTQAWLPCGQOSGP-VDYWNIEOSA-N	C29H40N2O9	HSP inhibitor	HSP90 AA1/AB1
164	97.92	lestaurtinib	UIARLYUEJFELEN-DMVVYWCZSA-N	C26H21N3O4	FLT3/GFR/JAK inhibitor	FLT3, NTRK 1/2/3, JAK2
**165**	**97.92**	**idarubicin**	**XDXDZDZNSLXDNA-TZNDIEGXSA-N**	**C26H27NO9**	**Topoisomerase inhibitor**	**TOP2A**
167	97.92	BI-2536	XQVVPGYIWAGRNI-JOCHJYFZSA-N	C28H39N7O3	PLK inhibitor	PLK 1/2/3, BRD4
170	97.89	alvocidib	BIIVYFLTOXDAOV-YVEFUNNKSA-N	C21H20ClNO5	CDK inhibitor	CDK 2/4/1/6/7/9/5/8, EGFR, PYGM
173	97.85	Merck60	ABZSPJVXTTUFAA-UHFFFAOYSA-N	C19H17N3O2S	HDAC inhibitor	HDAC 1/2
174	97.85	THM-I-94	MAUCONCHVWBMHK-UHFFFAOYSA-N	C21H23N3O5	HDAC inhibitor	HDAC 1/10/2/3/6/8
176	97.83	PIK-75	HFUAOBLWRUIXKP-MYHMWQFYSA-N	C17H17BrIN5O4S	DNA-PK/PI3K inhibitor	PIK3 CA/CB/CD/CG, PRKDC
177	97.82	dacinostat	BWDQBBCUWLSASG-MDZDMXLPSA-N	C22H25N3O3	HDAC inhibitor	HDAC 1/2/3/4/5/6/7/8/9
179	97.81	givinostat	YALNUENQHAQXEA-UHFFFAOYSA-N	C24H27N3O4	HDAC inhibitor	HDAC 2/1/3/4/5/6/7/8/9, IL 1B/1R2/6R
180	97.79	mocetinostat	HRNLUBSXIHFDHP-UHFFFAOYSA-N	C23H20N6O	HDAC inhibitor	HDAC 1/2/3/11
183	97.78	ER-27319	CFPDEQBPNROZDC-UHFFFAOYSA-N	C18H20N2O	SYK inhibitor	SYK
184	97.74	triptolide	DFBIRQPKNDILPW-KTGKZQHOSA-N	C20H24O6	RNAP inhibitor	CYP2C19, RELA
188	97.64	mitoxantrone	KKZJGLLVHKMTCM-UHFFFAOYSA-N	C22H28N4O6	Topoisomerase inhibitor	TOP2A, PIM1
191	97.61	bisindolylmaleimide-ix	DSXXEELGXBCYNQ-UHFFFAOYSA-N	C25H23N5O2S	CDK/PKC inhibitor	SIRT1, AKT1, GSK3B
194	97.5	pirarubicin	KMSKQZKKOZQFFG-YXRRJAAWSA-N	C32H37NO12	Topoisomerase inhibitor	TOP2A
195	97.5	JNJ-7706621	KDKUVYLMPJIGKA-UHFFFAOYSA-N	C15H12F2N6O3S	CDK inhibitor	CDK 1/2, AURK A/B
**215**	**97.21**	**wortmannin**	**QDLHCMPXEPAAMD-ZGSWIPFCSA-N**	**C23H24O8**	**PI3K inhibitor**	**PIK3 CA/CG/CD/R1, PLK 1/3, ATM**
**488**	**90.49**	**FCCP**	**BMZRVOVNUMQTIN-UHFFFAOYSA-N**	**C10H5F3N4O**	**MOP uncoupler**	

MOA: Mechanism of action; DNA-PK: DNA dependent protein kinase; RNAP: RNA polymerase; MOP: Mitochondrial oxidative phosphorylation

Table S5. Perturbagen Class analysis of drugs with opposed expression profiles for core prognostic gene set in CMap (count of all predicted drugs[1] or count of drugs with score>95[2]).

Rank	Score	Name	Perturbagens		
			Count[1]	Count[2]	Name (top 5)
PHARMACOLOGIC					
19	99.73	JAK inhibitor	5	2	JAK3-inhibitor-VI; cucurbitacin-i
29	99.59	MTOR inhibitor	16	16	WYE-125132; WYE-354; AZD-8055; PI-103; dactolisib
34	99.53	CDK inhibitor	12	9	Palbociclib; aminopurvalanol-a; purvalanol-a; CGP-60474; PHA-793887
36	99.52	IGF-1 inhibitor	3	2	BMS-754807; BMS-536924
41	99.48	PI3K inhibitor	16	9	KU-0060648; GDC-0941; PI-828; AS-605240; PIK-75
52	99.3	FLT3 inhibitor	6	3	TG-101348; lestaurtinib; midostaurin
67	99.15	HDAC inhibitor	20	19	ISOX; vorinostat; NCH-51; APHA-compound-8; entinostat
87	98.85	HSP inhibitor	7	3	HSP90-inhibitor; geldanamycin; NVP-AUY922
97	98.71	DNA-PK inhibitor	4	3	KU-0060648; NU-7441; VAMA-37
210	97.25	IKK inhibitor	5	4	TPCA-1; BMS-345541; IKK-16; BX-795
221	97.01	Topoisomerase inhibitor	16	11	Pidorubicine; idarubicin; mitoxantrone; pirarubicin; doxorubicin
260	96.42	EGFR inhibitor	14	4	WZ-3146; WZ-4-145; neratinib; tyrphostin-AG-1478
268	96.23	Aurora kinase inhibitor	14	5	reversine; alisertib; JWE-035; ENMD-2076; GSK-1070916
275	96.09	T-type calcium channel blocker	5	2	NNC-55-0396; mibefradil
280	95.95	HIF activator	4	2	VU-0418946-1; VU-0418947-2
315	95.17	VEGFR inhibitor	13	2	MAZ-51; tivozanib
GENETIC					
1	99.95	Cell Cycle Inhibition GOF	3	3	ccsbBroad304_00283; ccsbBroad304_05980; ccsbBroad304_00282
2	99.95	Lysine acetyltransferases LOF	3	2	CGS001-7994; CGS001-8202; CGS001-23522
75	99.01	Minor histocompatibility antigens LOF	3	1	CGS001-9322; CGS001-55746; CGS001-55127
82	98.89	C2 domain containing LOF	3	1	CGS001-7405; CGS001-25966; CGS001-22841
114	98.46	NFKB Activation GOF	7	3	ccsbBroad304_06542; ccsbBroad304_02048; ccsbBroad304_00259
137	98.21	Proteasome Pathway LOF	9	3	CGS001-5689; CGS001-5693; CGS001-5690
154	98.04	V type ATPases LOF	4	2	CGS001-527; CGS001-523; CGS001-9296
292	95.6	Ubiquitin-specific peptidases LOF	3	0	CGS001-219333; CGS001-7874; CGS001-9097

DNA-PK: DNA dependent protein kinase

Table S6. Mechanism of action (MOA) and Structures of overlapped drugs in both Cmap[1] and L1000FWD[2].

Drug	Tau[1]	Similarity[2]	Core structure	Literature report
CDK inhibitor				
**palbociclib**	**99.79**	**0.444**	**aminopyridine-pyridine[3,2-d]pyrimidine**	**In vitro**
**aminopurvalanol-a**	**99.65**	**0.327**	**phenylamino-purine**	
purvalanol-a	99.26	0.167	phenylamino-purine	In vitro
CGP-60474	98.1	0.111	phenylamino-pyrimidine-pyridine	
PHA-793887	98.03	0.105	amide bond-pyrazole-amide bond	
alvocidib	97.89	0.049	piperidine-chromanone-phenyl	In vitro
bisindolylmaleimide-ix	97.61	0.086	indole-pyrrol-indole	In vitro
JNJ-7706621	97.5	0.068	phenylamino-triazole-phenylbenzamide	
AT-7519	95.05	0.074	amide bond-pyrazole-amide bond	
MTOR inhibitor				
WYE-125132	99.05	0.198	morpholino-pyrazolo[3,4-d]pyrimidin-phenyl	
WYE-354	98.94	0.117	morpholino-pyrazolo[3,4-d]pyrimidin-phenyl	
AZD-8055	98.91	0.309	morpholino-pyrido[2,3-d]pyrimidine-phenyl	In vitro
PI-103	98.87	0.086	morpholino-pyrimidine-phenyl	In vitro
dactolisib	98.8	0.093	quinoline-imidazoquinoline-phenyl	In vitro
KU-0063794	98.52	0.210	morpholino-pyrido[2,3-d]pyrimidine-phenyl	
torin-2	97.43	0.235	pyridine-imidazoquinoline-phenyl	In vitro
torin-1	96.55	0.185	quinoline-imidazoquinoline-phenyl	In vitro
OSI-027	95.96	0.210	indole-imidazo[5,1-f][1,2,4]triazine-cyclohexane	In vitro
everolimus	95.77	0.043	macrocyclic lactone	In vitro
temsirolimus	95.64	0.056	macrocyclic lactone	Clinic
sirolimus	95.18	0.253	macrocyclic lactone	Clinic
LY-294002	91.95	0.253	morpholino-chromanone-phenyl	In vitro
tanespimycin		0.346	macrocyclic lactam	clinic
BRD-K18726304		0.321	benzothiazole-tetrahydrothieno[3,2-c]pyridine	
BRD-K38625260		0.315	benzothiazole-tetrahydrothieno[3,2-c]pyridine	
HDAC inhibitor				
ISOX	98.87	0.056	hydroxamic acids	
vorinostat	98.63	0.099	hydroxamic acids	In vitro
NCH-51	98.56	0.173	thiol	
APHA-compound-8	98.38	0.062	hydroxamic acids	
entinostat	98.11	0.099	benzamide	In vitro
WT-171	97.99	0.080	hydroxamic acids	
apicidin	97.82	0.043	cycic peptides	
dacinostat	97.82	0.080	hydroxamic acids	
givinostat	97.81	0.037	hydroxamic acids	
mocetinostat	97.79	0.049	benzamide	
Merck60	97.85	0.025	benzamide	
THM-I-94	97.85	0.093	hydroxamic acids	
panobinostat	97.34	0.056	hydroxamic acids	
pyroxamide	97.01	0.037	hydroxamic acids	
trichostatin-a	95.81	0.105	hydroxamic acids	
belinostat	95.57	0.043	hydroxamic acids	
scriptaid	94.71	0.123	hydroxamic acids	

HC-toxin	93.76	0.210	cyclic peptides	
NSC-3852	93.02	0.093	other	
PI3K inhibitor				
KU-0060648	98.84	0.086	morpholino-chromanone-anilin	
GDC-0941	98.48	0.130	morpholino-thieno[3,2-d]pyrimidine-indazole	
PI-828	98.38	0.340	morpholino-chromanone-anilin	
AS-605240	98.34	0.148	thiazolidinedione-quinoxaline	
PIK-75	97.83	0.062	imidazo[1,2-a]pyridine	In vitro
GSK-1059615	97.24	0.247	thiazolidinedione-quinoline-pyridine	
**wortmannin**	**97.21**	**0.302**	**wortmannin**	**Clinic**
ZSTK-474	96.12	0.210	morpholino-triazine-benzimidazole	In vitro
PIK-90	94.29	0.130	pyridine-amide bond-quinazoline	
BRD-K30836161		0.315	benzothiazole-thiophene	
BRD-K29506255		0.340	benzothiazole-thiophene	
EGFR inhibitor				
**WZ-3146**	**98.73**	**0.309**	**piperazine-phenylamino-pyrimidin**	
WZ-4-145	97.64	0.179	NA	
neratinib	91.45	0.130	phenylamino-quinoline	
tyrphostin-AG-1478	90.12	0.080	phenylamino-quinazoline	
FLT3 inhibitor				
TG-101348	98.73	0.105	phenylamino-pyrimidine-phenylamino	
lestaurtinib	97.92	0.123	staurosporine	
midostaurin	95.91	0.130	staurosporine	
IGF-1 inhibitor				
BMS-754807	98.7	0.198	aminopyrazol-pyrrolo[2,1-f][1,2,4]triazin-pyrrole	
BMS-536924	98.45	0.290	morpholino-benzimidazole-pyridine	
Exportin antagonist				
**7b-cis**	**98.31**	**0.333**	**phenyl-tetrazole**	
Topoisomerase inhibitor				
pidorubicine	98	0.080	anthracycline	
**idarubicin**	**97.92**	**0.333**	**anthracycline**	**Clinic**
mitoxantrone	97.64	0.099	anthracycline	In vitro
pirarubicin	97.5	0.074	anthracycline	Clinic
daunorubicin	97.09	0.074	anthracycline	In vitro
doxorubicin	97.03	0.154	anthracycline	In vitro
topotecan	95.7	0.123	camptothecin	Clinic
camptothecin	95.14	0.105	camptothecin	In vitro
ellipticine	95.03	0.049	camptothecin	In vitro
amsacrine	87.92	0.407	acridine	Clinic
teniposide	86.99	0.309	podophyllotoxin	Clinic
Aurora kinase inhibitor				
reversine	95.54	0.043	morpholino-phenylamino-imidazopyrimidine	
alisertib	95.43		phenylamino-pyrimidine-phenyl	
JWE-035	90.27	0.056	phenylamino-pyrimidine-phenylamino	
GSK-1070916	90.61	0.037	phenyl-pyrrolepyridine-pyrazole	
ENMD-2076	91.26		pyrimidine	
JAK inhibitor				
JAK3-inhibitor-VI	94.5	0.093	pyrrolo-indolone-pyridine	

cucurbitacin-i	93	0.191	triterpenoid	In vitro
Calcium channel blocker				
NNC-55-0396	94.12	0.136	quinoline	
mibefradil	93.2	0.265	quinoline	
niguldipine	58.23	0.309	other	
BRD-K65955264		0.377	other	
Mitochondrial oxidative phosphorylation uncoupler				
**FCCP**	**90.49**	**0.333**	**phenylhydrazone**	**In vitro**
